# SENP1-mediated NEMO deSUMOylation in adipocytes limits inflammatory responses and type-1 diabetes progression

**DOI:** 10.1038/ncomms9917

**Published:** 2015-11-24

**Authors:** Lan Shao, Huanjiao Jenny Zhou, Haifeng Zhang, Lingfeng Qin, John Hwa, Zhong Yun, Weidong Ji, Wang Min

**Affiliations:** 1 The First Affiliated Hospital, Center for Translational Medicine, Sun Yat-sen University, Guangzhou, China; 2 Department of Pathology, Interdepartmental Program in Vascular Biology and Therapeutics, Yale University School of Medicine, 10 Amistad St, New Haven, Connecticut 06520, USA; 3 Department of Internal Medicine and Section of Cardiology, Yale University School of Medicine, New Haven, Connecticut 06510, USA; 4 Department of Therapeutic Radiology, Yale University School of Medicine, New Haven, Connecticut 06510, USA

## Abstract

Adipocyte dysfunction correlates with the development of diabetes. Here we show that mice with a adipocyte-specific deletion of the SUMO-specific protease SENP1 gene develop symptoms of type-1 diabetes mellitus (T1DM), including hyperglycaemia and glucose intolerance with mild insulin resistance. Peri-pancreatic adipocytes from SENP1-deficient mice exhibit heightened NF-κB activity and production of proinflammatory cytokines, which induce CCL5 expression in adjacent pancreatic islets and direct cytotoxic effects on pancreatic islets. Mechanistic studies show that SENP1 deletion in adipocytes enhances SUMOylation of the NF-κB essential molecule, NEMO, at lysine 277/309, leading to increased NF-κB activity, cytokine production and pancreatic inflammation. We further show that NF-κB inhibitors could inhibit pre-diabetic cytokine production, β-cell damages and ameliorate the T1DM phenotype in SENP1-deficient mice. Feeding a high-fat diet augments both type-1 and type-2 diabetes phenotypes in SENP1-deficient mice, consistent with the effects on adipocyte-derived NF-κB and cytokine signalling. Our study reveals previously unrecognized mechanism regulating the onset and progression of T1DM associated with adipocyte dysfunction.

## Abstract (web summary)

Pro-inflammatory NF-κB signalling is regulated by protein sumoylation. Here the authors show that lack of the desumoylating protease SENP1 in fat tissue induces NF-κB activity and inflammation in peri-pancreatic adipocytes, leading to symptoms of type 1 diabetes in mice.

## Graphical abstract

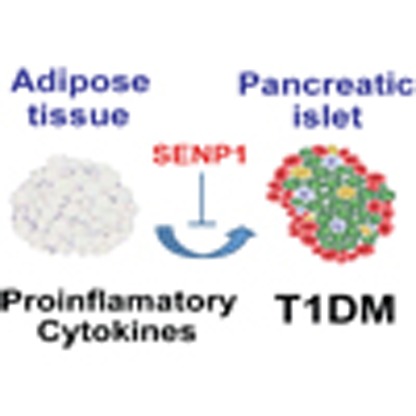

One of the key phenotype of type-1 diabetes mellitus (T1DM) is characterized by the autoimmune-mediated destruction of the pancreatic β cells. The autoimmune attack on the pancreatic β cells can be detected years before clinical onset of T1DM-related autoantibodies in the blood^1^,^2^,^3^. Prospective studies of T1DM have established that T1DM exhibits the elevated levels of inflammatory markers before diagnosis of diabetes. There is a significant elevation in the interleukin-6 (IL-6), C-reactive protein (CRP), tumour necrosis factor-alpha (TNF-α) and IL-1β levels in preclinical diabetes samples. These cytokines can induce β-cell death in T1DM^4^. The autoimmunity may occur because of instructive immune responses^2^,^3^. Persistent elevation of proinflammatory cytokines in the body can be defined as a risk factor that, either alone or in combination with other environment factors, may predispose to the loss of self-tolerance and the onset of T1DM-related autoantibodies.

Adipose tissue functions are closely linked to development of diabetes, particularly type-2 diabetes mellitus (T2DM). The roles of adipose tissue in glucose metabolism, lipodystrophy and insulin resistance are well known^5^,^6^. Recent studies indicate that adipose tissue is not simply the organ that stores fat and regulates lipid metabolism but also is the largest endocrine organ with immune functions^5^. Adipocytes produce several mediators, such as adiponectin, resistin, IL-6, TNF-α, leptin, monocyte chemotactic protein-1 (or CCL2) and IL-1β, all of which participate in the immune response as proinflammatory mediators. It is reported that adipocytes are responsible for almost one-third of the IL-6 concentration in diabetic patients^5^.

One of the critical activators of inflammatory genes is NF-κB^7^,^8^. Experimental evidences have suggested that SUMOylation components regulate NF-κB signalling and transcriptional activity^9^,^10^. NEMO is part of the cytoplasmic IκBα kinase (IKK) complex that is critical for NF-κB activation not only by the majority of extracellular signals, including TNF-α and IL-1β, but also in response to many genotoxic stress agents. NEMO is SUMO1 modified on K277/K309 with the help of SUMO E1/E2 and an E3 (PIASy)^11^. The reverse SUMOylation of NEMO by SUMO endopeptidases (SENPs) plays an important role in inhibiting nuclear factor kappa-light-chain-enhancer of activated B cells (NF-κB) activity and NF-κB-dependent transcriptional activation^12^,^13^.

As a post-translational modification, SUMOylation is involved in various cellular processes, such as nuclear–cytosolic transport, transcriptional regulation, apoptosis, protein stability, response to stress and cell cycle progression. SUMOylation is a dynamic process that is mediated by activating, conjugating and ligating enzymes, and is readily reversed by a family of deSUMOylating proteases SENPs^14^,^15^. SENP1 is a deSUMOylating protease that deconjugates a large number of SUMOylated proteins^14^. Previously, we and others have observed that a global deletion of SENP1 causes deficient haematopoiesis and prenatal lethality^16^,^17^, therefore excluding further studies on the role of SENP1 in inflammation and diabetes. Interestingly, several components in SUMOylation have been identified as candidate genes implicated in T1DM susceptibility^18^,^19^. However, the underlying mechanism by which SUMOylation pathway regulates T1DM, and whether or not the SUMOylation signalling play a role in adipocyte is unclear. Therefore, in the present study, we attempt to address whether persistent protein SUMOylation in adipocytes affects T1DM onset and progression.

We have created genetically modified mice with an adipocyte-specific deletion of SENP1 with three different adipocyte-specific Cre deleter lines. Our present data show that adipocyte-specific SENP1-deficient mice are viable but develop the major phenotypes of T1DM, including hyperglycaemia, glucose intolerance, increases in cytotoxic T cells and autoantibody production. This diabetic phenotype observed in SENP1-deficient mice is associated with high SUMOylation of NEMO, a key component regulating NF-κB, in pancreatic adipocytes. Our data suggest that NEMO SUMOylation in adipocytes is a strong risk factor in T1DM.

## Results

### SENP1-deficient mice exhibit T1DM phenotype

To investigate possible function of SENP1 in inflammation and diabetes, we created genetically modified mice with an adipocyte-specific deletion of SENP1 with three different deleter lines carrying the Cre recombinase driven by the *Adiponectin*, *PdgfR*α or *aP2/Fabp4* gene promoter (named as SENP1-AdipoqKO, SENP1-PdgfRKO and SENP1-aP2KO, respectively; Supplementary Fig. 1A). While the PdgfRα-Cre is predominantly expressed in bipotent progenitor adipocytes (white and beige adipocytes)^20^,^21^, the Adipoq-Cre has high efficiency and specificity in mature adipocytes^22^,^23^. The aP2*-*Cre has a moderate efficiency in adipocytes^24^,^25^,^26^ with variable degrees of target gene recombination in macrophages^27^ or capillary endothelium in a floxed gene locus-dependent manner^22^,^23^.

SENP1 expression (messenger RNA (mRNA) and protein) was diminished in isolated adipocytes from interscapular brown adipose tissue, perigonadal white adipose tissue and peri-pancreatic adipose tissue (PAT) of SENP1-AdipoqKO, SENP1-PdgfRKO (with less efficiency in brown adipose tissue) and SENP1-aP2KO mice compared with the control SENP1^lox/lox^ (Ctrl). In contrast, SENP1 expression was intact in isolated macrophages (CD11b^+^F4/80^+^) and pancreatic islets from these mice (Supplementary Fig. 1B–C). Moreover, enhanced green fluorescent protein was detected in PATs but not in pancreatic islets when aP2-Cre deleter mice mating with mT/mG reporter mice (Supplementary Fig. 1D).

**Figure 1:**
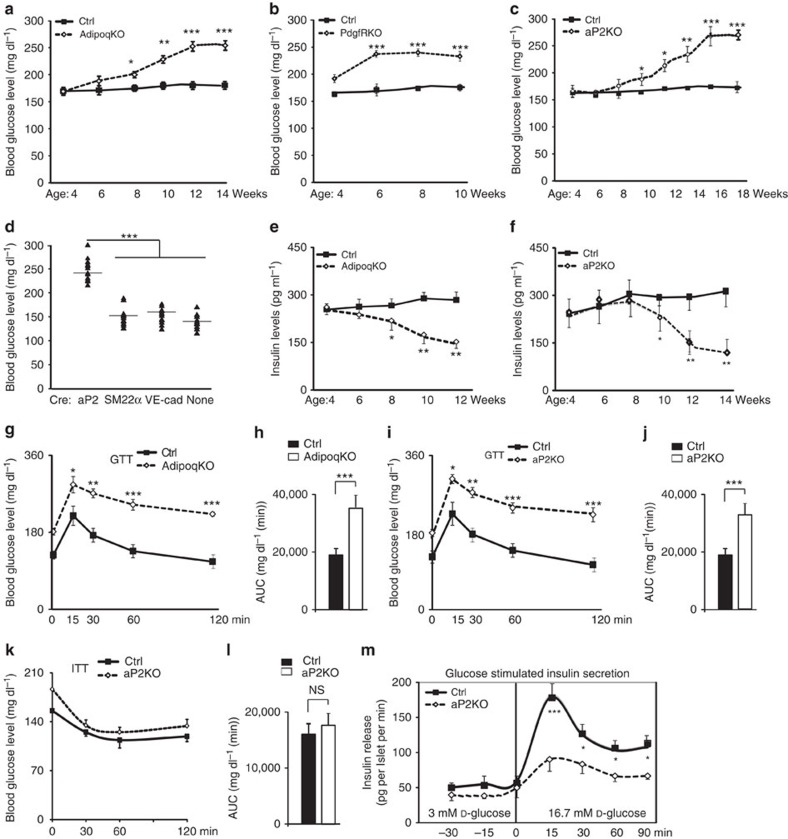
Adipocyte-specific SENP1-deficient mice exhibit type-1 diabetic phenotype. (**a**–**c**) Glucose levels in SENP1-AdipoqKO (**a**), SENP1-PdgfRKO (**b**), SENP1-aP2KO (**c**) mice were compared with Ctrl. Glucose levels were measured at the ages of 4, 6, 8, 10, 12, 14, 16 and 18 weeks (male; *n*=11 each group). (**d**) Glucose levels in Ctrl (male; *n*=14), SENP1-aP2KO (male; *n*=14), SENP1-smKO (male; *n*=13) and SENP1-ecKO (male; *n*=14) were compared at the age of 14 weeks. (**e**,**f**) Insulin levels, insulin levels in Ctrl and SENP1-AdipoqKO (**e**) or Ctrl and SENP1-aP2KO mice (**f**) were measured at the ages of 4, 6, 8, 10, 12 and 14 weeks (male; *n*=7 each group). (**g**–**j**) GTT assay. 14-week-old mice were fasted overnight, followed by an intraperitoneal injection of glucose (1 g kg^−1^ body weight). Blood glucose levels were determined by an electronic glucometer at various times post-injection. (**g**) Ctrl (male; *n*=11) and SENP1-AdipoqKO (male; *n*=10) mice; (**i**) Ctrl (male; *n*=12) and SENP1-aP2KO (male; *n*=13) mice. Area under curve (AUC) in 120 min was calculated (**h**,**j**). (**k**,**l**) ITT assay. 14-week-old mice were fasted overnight, followed by an intraperitoneal injection of insulin (0.75 U kg^−1^ body weight). Blood glucose levels were determined at various times post-injection and AUC in 120 min was calculated. Ctrl (*n*=9, male) and SENP1-aP2KO mice (male; *n*=11). (**m**) Glucose-stimulated insulin release assay. Pancreatic islets isolated from 14-week-old Ctrl (*n*=10, male) and SENP1-aP2KO mice (*n*=10, male) were stimulated with 3 mM glucose for 30 min, followed by stimulation with 16.7 mM glucose for 90 min. Insulin secreted into the media was measured by ELISA and calculated as amount of insulin release per islet. The two-tailed Student’s unpaired *t*-test was used for the statistical analysis. All results are shown as means±s.e.m. *P<0.05; **P<0.01; ***P<0.001. Ctrl, control; NS, non-significance.

SENP1-AdipoqKO, SENP1-PdgfRKO and SENP1-aP2KO mice were viable after birth. However, these adipocyte-specific SENP1-deficient mice all developed a diabetic phenotype (Fig. 1a–c). An age-dependent increase in glucose levels was observed in SENP1-AdipoqKO, SENP1-PdgfRKO and SENP1-aP2KO mice compared with the control SENP1^lox/lox^ mice with an early onset of diabetes in SENP1-PdgfRKO at the age of 6 weeks (blood glucose level >225 mg dl^−1^); this earlier onset of hyperglycaemia is consistent with the high efficiency of PdgfRα-Cre in adipocyte progenitors. We observed similar phenotypic changes in both male (Fig. 1a–c) and female with or without fasting, and we focused on male mice for further studies. We measured glucose levels in SENP1-smKO and SENP1-ecKO mice, in which the SENP1 gene was specifically deleted in smooth muscle cells/pericytes by SM22α-Cre (SENP1-smKO) and in vascular endothelial cells by VE-cadherin-Cre (SENP1-ecKO), respectively^28^,^29^. Unlike SENP1-aP2KO mice, SENP1-smKO and SENP1-ecKO mice did not develop a high-glucose phenotype even at the age of 14 weeks (Fig. 1d).

Insulin levels in plasma were measured at various ages, and insulin secretion in SENP1-AdipoqKO and SENP1-aP2KO mice was significantly declined after the ages of 8 and 10 weeks, respectively (Fig. 1e,f). Glucose tolerance test (GTT) and insulin tolerance test (ITT) were performed at the age of 14 weeks to evaluate diabetes and insulin resistance. The level of hyperglycaemia observed with the GTT was prolonged in SENP1-AdipoqKO and SENP1-aP2KO mice compared with Ctrl mice (Fig. 1g–j). However, similar insulin responses were observed between SENP1-deficient mice and Ctrl in the ITT assays (Fig. 1k,l for aP2KO mice). We then performed glucose-stimulated insulin assay with isolated islets. Glucose-stimulated insulin secretion from SENP1-deficient islets was significantly reduced (Fig. 1m). These data suggest that the high glucose level in SENP1-deficient mice was due to reduced insulin secretion.

**Figure 2:**
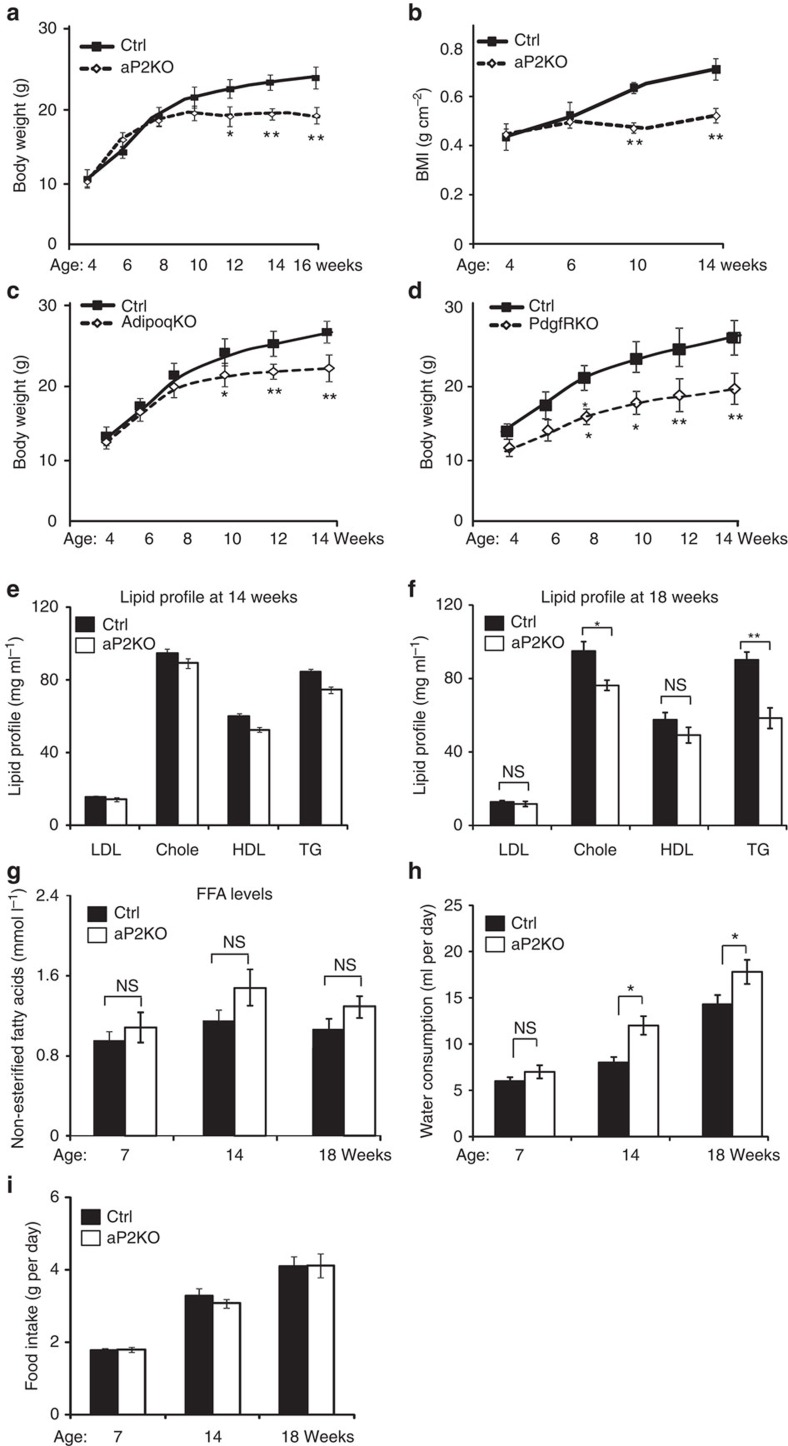
Body weight, lipid profiles, water consumption and food intake in SENP1-deficient mice. (**a**) Body weight in Ctrl and SENP1-aP2KO mice was measured at the indicated ages (*n*=6 male in each group). (**b**) Body mass index (BMI) was calculated as body weight (**g**) divided by the square of the anal-nasal length (cm^2^) at the indicated ages. (**c**,**d**) Body weights in Ctrl, SENP1-adipoqKO and SENP1-PdgfRKO mice were measured at the indicated ages (male; *n*=8 for each group). (**e**,**f**) Mean blood lipoprotein levels in Ctrl and SENP1-aP2KO mice at the ages of 14 and 18 weeks (male; *n*=8 for each group). (**g**) Mean blood FFA levels in Ctrl and SENP1-aP2KO mice at indicated age (male; *n*=8 for each group). (**h**,**i**) Water consumption and mean daily food intake were measured in Ctrl and SENP1-aP2KO at indicated ages (male; *n*=8 for each group). The two-tailed Student’s paired *t*-test was used for the statistical analysis. All data are presented as means±s.e.m., *n*=8. *P<0.05; **P<0.01; Ctrl, control; NS, non-significance.

The body weight and body mass are associated with the development of diabetes and increases in glucose levels. All SENP1-deficient mice had lower body weights compared with the control mice after onset of diabetes (Fig. 2a–d). However, adipocyte-specific SENP1-deficient mice exhibited normal lipid profiles up to the age of 14 weeks, including total cholesterol, low-density lipoprotein, high-density lipoprotein (HDL), triglyceride (TG) and free fatty acid (FFA). Cholesterol and TG, but not HDL or FFA, were slightly reduced in SENP1-aP2KO mice after the age of 18 weeks (Fig. 2e–g). Water consumption and food-intake monitoring showed that SENP1-aP2KO mice had increased water consumption with similar dairy food uptake compared with Ctrl mice (Fig. 2h–i). Taken together, the assays described above suggest that type-1-like diabetic phenotypes developed in the adipocyte-specific SENP1-deficient mice.

### SENP1 deficient mice show autoimmune mediated damages

**Figure 3:**
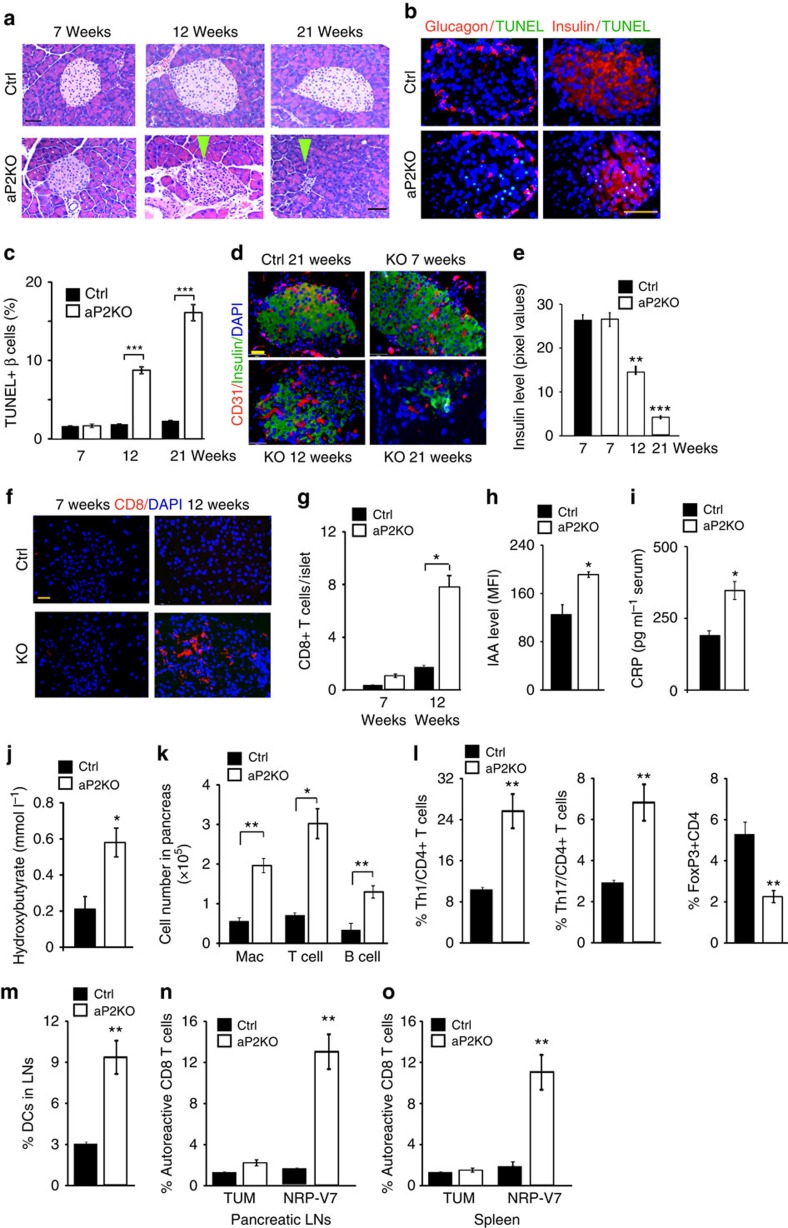
Characterization of β-cell apoptosis and autoimmune responses in SENP1-deficient mice. (**a**) Haematoxylin and eosin staining for overall morphology of the pancreases from Ctrl and SENP1-aP2KO mice was compared. Representative images of the pancreases from Ctrl (*n*=6, male) and SENP1-aP2KO mice (*n*=6, male) at the ages of 7, 12 and 21 weeks are shown. Three sections from each mouse. Data are representative for three independent experiments. Arrowheads indicate structural changes in the islets of SENP1-aP2KO mice. (**b**,**c**) β-Cell apoptosis. TUNEL *in situ* cell death staining together with α-cell marker glucagon and β-cell marker insulin. Representative images from Ctrl (*n*=6, male) and SENP1-aP2KO mice (*n*=6, male) at the age of 12 weeks are shown in **b** and TUNEL-positive β cells in 7, 12 and 21 weeks were quantified in **c**. Data are means±s.e.m. of five sections from each islet. Data are representative for three independent experiments. (**d**,**e**) CD31 and insulin co-staining from Ctrl mice (*n*=6, male) and SENP1-aP2KO mice (*n*=6, male) (**d**) and insulin level were calculated by Image J software (**e**). (**f**,**g**) T-cell infiltration. Representative images are anti-CD8 staining (red) of Ctrl (*n*=6, male)and SENP1-aP2KO mice (*n*=6, male) at the ages of 7 and 12 weeks (**f**) and numbers of CD8^+^ T cells invading into the islet of Ctrl and SENP1-aP2KO mice were quantified (**g**). Data are means±s.e.m. of five sections from each islet. Data are representative for three independent experiments. (**h**–**j**) Autoimmune markers. Insulin autoantibody (IAA), CRP and β-hydroxybutyrate were determined using ELISA. (**k**) Number of macrophages, T cells and B cells in pancreases were measured and quantified by FACS at the age of 12 weeks. (**l**) T-cell subset distribution. Percentages of Th1 (IFN-γ^+^CD4^+^) effector T cells, Th17 (IL-17 A^+^CD4^+^) effector T cells and regulatory T cells (CD4^+^CD25^+^Foxp3^+^) in pancreatic lymph nodes of Ctrl and SENP1-aP2KO mice were detected by FACS and quantified. (**m**) Percentage of DCs in pancreatic lymph nodes was quantified by FACS at the age of 12 weeks. (**n**,**o**) CD8^+^ T cells isolated from pancreatic lymph nodes (**n**) and spleen (**o**) of 12-week-old Ctrl and aP2KO mice were stained with TUM (Ctrl) and islet autoantigen (NRP-V7)-specific tetramers followed by quantifications by FACS. Scale bar, 20 μm (in all images). The two-tailed Student’s paired *t*-test was used for the statistical analysis. All data are presented as means±s.e.m. from six paired samples of Ctrl and SENP1-aP2KO male mice at each age. *P<0.05; **P<0.01; ***P<0.001. Ctrl, control; DS, dendritic cell; KO, knockout; LN, lymph node.

Insulin-dependent diabetes mellitus results from T-cell-mediated destruction of insulin-producing pancreatic islet β cells^1^,^2^,^3^. To determine the pathogenesis of diabetes in SENP1-deficient mice, we examined pancreatic development and pathology by haematoxylin and eosin stain and immunostaining for pancreatic β-cell number, mass and morphology changes, as well as immune cell invasion, indicative of insulitis. At early ages before the onset of hyperglycaemia, no significant differences were detected in pancreatic mass, β-cell number between the Ctrl and SENP1-deficient mice. However, the structures of pancreatic islets in SENP1-AdipoqKO, SENP1-PdgfRKO and SENP1-aP2KO mice were drastically disrupted with increased TdT-mediated dUTP nick end labelling (TUNEL)-positive apoptosis of β cells after onset of diabetes and more severe at late stages (Fig. 3a–c for SENP1-aP2KO; Supplementary Fig. 2 for SENP1-AdipoqKO and SENP1-PdgfRKO). There was a sharp decline in insulin staining and CD31^+^ vascular area in the islets in SENP1-aP2KO mice (Fig. 3d with quantifications in Fig. 3e), indicating a loss of function in the β cell with disrupted vasculatures. These data provide direct evidence in support of apoptosis as the mechanism of β-cell death in SENP1-deficient mice.

Immune cell-specific effector T-cell invasion in the pancreatic tissue attacks the islets and induces cell death. We observed an accumulation of white blood cells invasion into the islets in SENP1-deficient mice. In T1DM, β cell and islets are attacked by activated cytotoxic T cells^30^,^31^. The section staining in the pancreases demonstrated that CD8^+^ T cells were absent from the islets of Ctrl mice. No pancreatic infiltrations of immune cells were detected in the SENP1-aP2KO mice under the age of 7 weeks. However, CD8^+^ T cells infiltrated into the islets of SENP1-aP2KO mice by the age of 12 weeks (Fig. 3f,g). Similar results were observed for CD4^+^ T cells (Supplementary Fig. 3A–B). These data strongly suggest a T-cell dominant mechanism for β-cell destruction.

The pre-diabetic phase of the disease usually begins several years before the clinical onset of the disease. During this phase, autoantibodies, mainly insulin autoantibody (IAA), are directed against β cells. IAA levels, along with other clinical information, aid in the diagnosis of T1DM^32^,^33^. CRP and β-hydroxybutyrate are important clinical parameters for the diagnosis of diabetes^34^. Individuals who develop diabetes typically have higher CRP levels. Beta-hydroxybutyrate is classified as ketones that are produced in the body when the body loses its ability to absorb glucose properly. SENP1-aP2KO mice had increased levels of IAA in the blood as measured by enzyme-linked immunosorbent assay (ELISA) with an IAA-specific antibody. Greater levels of CRP and β-hydroxybutyrate were also observed in the blood of SENP1-aP2KO mice (Fig. 3h–j). All of the above data support the appearance of typical T1DM in SENP1-aP2KO mice.

We then fully characterized the immune system in the SENP1-deficient mice. We transplanted skin from mice (BALB/C) to Ctrl SENP1^lox/lox^ and SENP1-aP2KO mice (C57BL/6 background). Severe skin-rejection response appeared in both Ctrl and SENP1-aP2KO mice at an equal level, indicating SENP1-aP2KO mice are not immunodeficient (Supplementary Fig. 3C). We examined the immune cell populations in pancreas, pancreatic lymph nodes and spleen of SENP1-deficient mice. The distribution of immune cells in the pancreases was determined by fluorescence-activated cell sorting (FACS) analyses with specific surface markers. MHCII^+^CD11b^+^CD11c^−^ macrophages, CD3e^+^ T cells and CD19^+^ B cells were markedly increased in the pancreases of SENP1-aP2KO versus the Ctrl mice (Supplementary Fig. 3D for FACS and Fig. 3k for quantifications). Subset T cells play a key role in regulation of inflammation and immune tolerance^35^. We observed that Th1 (IFN-γ^+^CD4^+^) and Th17 (IL-17^+^CD4^+^) effector T-cell subsets expanded concomitant with reduction of regulatory T-cell subset (CD4^+^CD25^+^Foxp3^+^) in the pancreatic lymph nodes of SENP1-aP2KO mice (Supplementary Fig. 3E–G for FACS and Fig. 3l for quantifications). We also observed an increase in activated dendritic cells in the lymph node of pancreases (Fig. 3m). Moreover, an autoantigen NRP-V7-positive CD8^+^ T cells, targeting a peptide from islet-specific glucose-6-phosphatase catalytic subunit-related protein^36^, were detected in the pancreatic lymph nodes and the spleen of SENP1-aP2KO mice at the age of 12 weeks (Supplementary Fig. 3H for FACS and Fig. 3n,o for quantifications). All of the events suggest autoimmune responses occur in the pancreases of SENP1-deficient mice, consistent with that T1DM is an autoimmune disease in which insulin deficiency is a consequence of immunological destruction of the pancreatic β cells^1^,^2^,^3^.

### PATs secret cytokines

**Figure 4:**
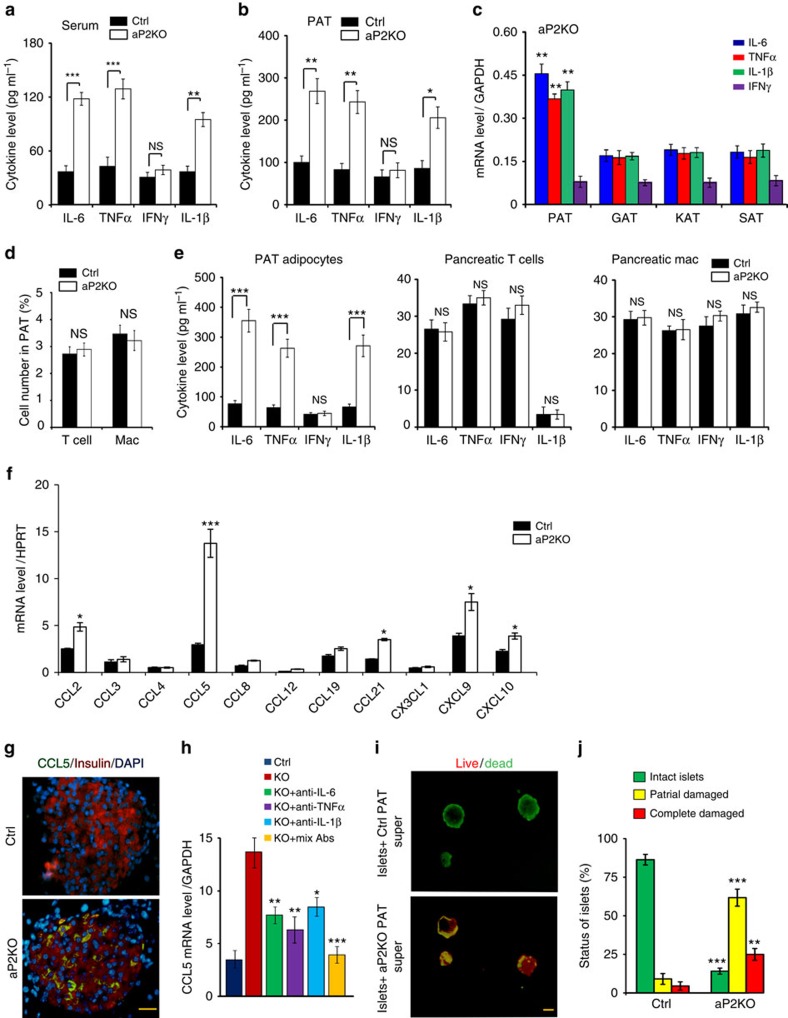
SENP1 deletion augments PAT inflammation and islet immunogenicity before onset of diabetes. (**a**,**b**) Cytokine expression, concentrations of IL-6, TNF-α, IFN-γ and IL-1β proteins in serum (**a**) and PATs (**b**) were measured by ELISArray kits in six pairs of age-matched Ctrl and SENP1-aP2KO mice at the age of 7 weeks, *n*=6, male. (**c**) Cytokine expression in adipose tissues. Transcript levels of IL-6, TNF-α, IFNγ and IL-1β in pancreatic, gonadal, peri-renal and subcutaneous inguinal adipose tissue (PAT, GAT, KAT and SAT, respectively) were quantified by quantitative PCR with reverse transcription (qRT–PCR). GAPDH was used for normalization. *n*=6 male mice at the age of 7 weeks. (**d**) Total T cells (CD3^+^) and macrophages (CD11b^+^F4/80^+^) in PAT of Ctrl (*n*=6, male) and SENP1-aP2KO (*n*=6, male) mice were detected by FACS at the age of 7 weeks. Quantifications of total T cells and macrophages are presented. (**e**) Protein levels of IL-6, TNF-α, IFN-γ and IL-1β levels present in culture supernatant of isolated PAT adipocytes, T cells and macrophages were detected with ELISA after 24 h culture. Data are means±s.e.m. *n*=6, male. All data are means±s.e.m. from *n*=6 male mice per group. The two-tailed Student’s paired *t*-test was used for the statistical analysis. *P<0.05; **P<0.01; NS, non-significance. (**f**–**h**) CCL5 expression. mRNAs of chemokines in islets which isolated from Ctrl (*n*=10, male) and SENP1-aP2KO mice (*n*=10, male ) were quantified by TaqMan PCR with normalization by HPRT (**f**). Sections of pancreases from Ctrl and SENP1-aP2KO mice were co-immunostaining with CCL5 (green) and β-cell marker insulin (red). Representative images of the pancreases from Ctrl and SENP1-aP2KO mice at the age of 7 weeks are shown (**g**). Scale bar, 20 μm. Three sections from each mice, *n*=6 male mice per group at each age. (**h**) Mice pancreatic islets (100 islets per well) were incubated with culture supernatant of adipocyte collected from SENP1-aP2KO mice at the age of 7 weeks with or without IL-6, TNF-α or IL-1β neutralization antibody as indicated. After 24 h, mRNA level of CCL5 were quantified by qRT–PCR. *n*=6 male mice per group at each age. (**i**,**j**) Direct effects of culture supernatant of peri-pancreatic adipocytes on islets. Mice pancreatic islets were isolated from Ctrl mice. Islets (100 islets per well) were cultured with culture supernatant of peri-pancreatic adipocytes from Ctrl, SENP1-aP2KO mice at the age of 5 weeks. After 72-h incubation, islet structure and apoptosis were monitored by Live/Dead viability/Cytotoxicity kit and detected by fluorescence microscopy. Representative images from each group are shown. Scale bar, 200 μm. Data are representative for three independent experiments. Three images from each mice, *n*=6 male mice per group. (**j**) Quantifications of intact islets (green), partial damaged islets (yellow) and completed damaged islets (red). Total 300 islets from each group were counted. The two-tailed Student’s paired *t*-test was used for the statistical analysis. All data are means±s.e.m., *P<0.05; **P<0.01; ***P<0.001. Abs, antibiodies; Ctrl, control; DAPI, 4′,6-diamidino-2-phenylindole; HPRT, hypoxanthine-guanine phosphoribosyltransferase; NS, non-significance; .

The proinflammatory cytokines overproduction in early phase of disease could serve as a main reason for immunologic tolerance break and immune cell expansion in diabetes^1^,^2^,^3^. We found that the circulating proinflammatory cytokines IL-6, TNF-α and IL-Iβ, but not IFN-γ, were significantly elevated in the SENP1-deficient mice before the onset of diabetes at the age of 7 weeks (Fig. 4a). However, the levels of circulating cytokines might be too low to induce systemic autoimmune responses. Consistently, SENP1-deficient mice had normal cardiac function and vascular reactivity. These mice exhibit increased systemic cytokines and cardiac myocarditis at the age of 14 weeks or older, indicating a prevalence of diabetic phenotype in the SENP1-deficient mice.

Adipose tissue has been recognized as the largest endocrine organ and produces a large amount of cytokines^5^. We reasoned that PATs might express high levels of cytokines to induce local (pancreas) inflammatory responses and diabetic phenotype in SENP1-deficient mice. Indeed, PATs from SENP1-aP2KO mice expressed high levels of IL-6, TNF-α and IL-1β at the age of 7 weeks (Fig. 4b; Supplementary Fig. 4A). We also compared cytokine transcripts expressed in the adipocytes from PATs, subcutaneous (inguinal) and gonadal and peri-renal adipose tissues at the age of 7 weeks. Ctrl mice expressed little basal cytokines in all adipose tissues. We did not observe significant differences for SENP1 deletion in different adipose depots. However, SENP1 deletion strongly enhanced gene expression of proinflammatory cytokines in the PATs compared with other adipose depots (Fig. 4c). To exclude the impact of macrophages or T cells affects the cytokine concentration, we examined immune cell numbers and cell-type-specific cytokine expression in the pancreas and PATs. We did not detect significant differences in cell numbers of macrophages and T cells in PATs (Fig. 4d) or pancreas of Ctrl and SENP1-aP2KO mice at the age of 7 weeks. Moreover, expression levels of IL-6, TNF-α and IL-1β by isolated pancreatic T cells and macrophages were >10-fold lower than isolated PAT adipocytes as measured by ELISA (Fig. 4e). Similar results were obtained from measuring intracellular cytokine levels in adipocytes, T cells and macrophages within PATs by FACS on intracellular staining of single cells (Supplementary Fig. 4B), suggesting that peri-pancreatic adipocytes primarily contribute to cytokine production in SENP1-aP2KO mice before the onset of diabetes. Of note, we detected macrophages infiltration into the pancreases of SENP1-aP2KO mice at later phases (14 weeks), and these macrophages expressed high levels of proinflammatory cytokines (Supplementary Fig. 4C–D).

We reasoned that the local high concentrations of cytokines might induce chemokine expression in adjacent pancreatic islets to recruit immune cells. It is known that CCL5 (RANTES, the CCR5 ligand) can direct recruitment of immune cells to inflammation sites or to autoimmune targets that may contribute to type-1 diabetic development^37^. We analysed gene expression of chemokines that had been reported to be expressed in the pancreas. We found that CCL5 was strongly upregulated in the pancreatic islets of SENP1-aP2KO mice at the age of 7 weeks before disease the onset. CCL2, CCL21, CXCL19 and CXCL10 were weakly upregulated in pancreatic islets of SENP1-aP2KO mice (Fig. 4f). Expression of CCL5 in islet β cells was confirmed by co-staining of CCL5 with insulin (Fig. 4g). However, upregulation of CCL5 was not detected in other tissues of SENP1-aP2KO mice at the age of 7 weeks (Supplementary Fig. 4E), suggesting a specific effect of PAT-derived cytokines on adjacent islets. This is consistent with human protein expression database that CCL5 is only detected in pancreas but not in other tissues except blood and immune cells (GeneCards; http://www.genecards.org/cgi-bin/carddisp.pl?gene=CCL5). We then directly tested whether proinflammatory cytokines secreted from PATs could induce CCL5 expression in isolated islets. CCL5 mRNA in islets was significantly upregulated by conditional media of SENP1-aP2KO pancreatic adipocytes, and this upregulation was reduced by a neutralizing antibody to IL-6, TNF or IL-1β, and blunted by a combination of the three antibodies (Fig. 4h). These data suggest that high levels of local proinflammatory cytokines secreted from PATs induces CCL5 expression in islets, which in turn recruits immune cells into pancreas, causing damages of β cells.

We also tested a direct effect of adipocyte-derived cytokines on the pancreatic islets. To this end, isolated normal mouse pancreatic islets were incubated with culture supernatant of adipocytes from Ctrl or SENP1-aP2KO, and islet structure and apoptosis were monitored by Live/Dead viability/Cytotoxicity assays. We found that culture supernatant from adipocytes of SENP1-aP2KO strongly induced β-cell death and pancreatic disruption (Fig. 4i,j), suggesting that cytokines from peri-pancreatic adipocytes of SENP1-aP2KO mice have direct cytotoxic effects on pancreatic islet.

Taken together, these results indicate that high local concentrations of cytokines derived from PATs not only upregulate CCL5 expression and CCL5-mediated infiltration of immune cells in pancreas but also directly induce cytotoxic damages on pancreatic islets.

### SENP1 deletion augments NF-κB dependent inflammation

**Figure 5:**
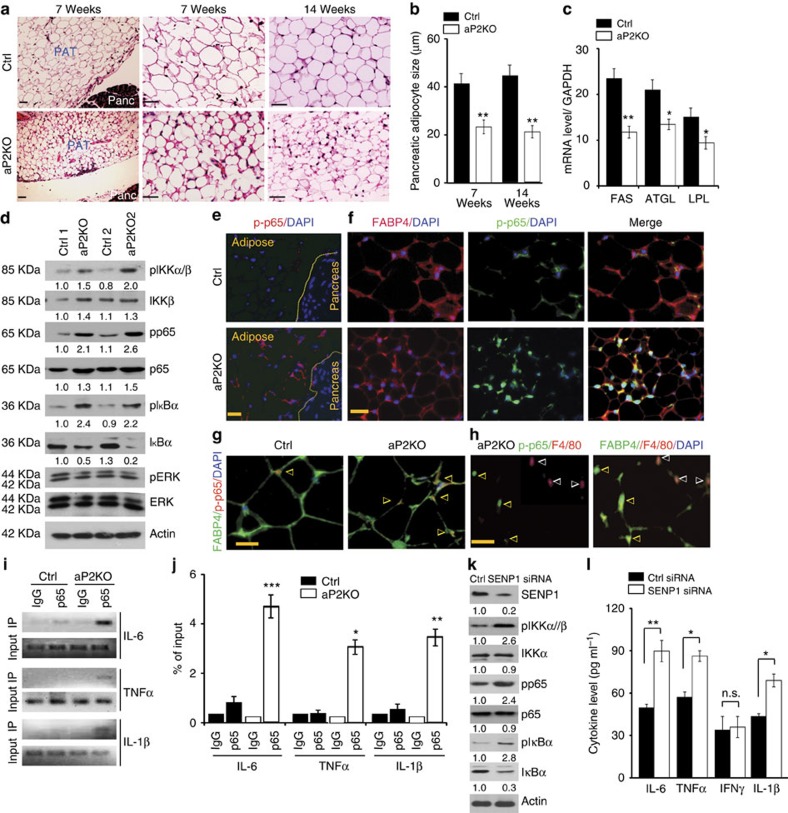
SENP1 deletion alters pancreatic adipocyte phenotype and augments NF-κB-dependent inflammation. (**a**,**b**) Pancreatic adipose was collected from 7 and 14 weeks old Ctrl and SENP1-aP2KO male (male, *n*=6). Morphology was visualized by haematoxylin and eosin stain staining. Scale bar, 20 μm (**a**). Cell sizes were quantified in (**b**). Three sections from each adipose tissue. Data are representative for three independent experiments. (**c**) Transcript levels of adipocyte differentiation markers (fatty acid synthase, adipose triglyceride lipase and lipoprotein lipase) in PATs were quantified by quantitative PCR with reverse transcription with GAPDH for normalization. *n*=6, male for each group. (**d**) Increased IKK-NF-κB activities in SENP1-aP2KO PATs. A representative western blot was from Ctrl and SENP1-aP2KO mice at the age of 7 weeks. Data are representative from three independent experiments is shown (*n*=6, male). Relative protein levels were quantified from three blots by taking Ctrl as 1.0 (*n*=6, male). (**e**–**h**) NF-κB activation is specifically detected in PATs of SENP1-aP2KO mice. (**e**) Phosphor-p65 staining (red) in PATs but not in pancreas. (**f**) Co-immunostaining of phosphor-p65 (green) and adipocyte marker FABP4 (red). (**g**) High-power images show co-staining of phosphor-p65 (red) in the nucleus of FABP4^+^ adipocytes (green). (**h**) Co-staining of phosphor-p65 (green) with APC-conjugated adipocyte marker FABP4 (green; yellow arrowheads), but not with macrophage marker F4/80 (red; white arrowheads). Representative images are from one of three sections from SENP1-aP2KO PATs and *n*=3 male mice. Scale bar, 20 μm. (**i**,**j**) ChIP assay. ChIP assays with p65/RelA antibody were performed in the adipocyte isolated from Ctrl (*n*=6, male) and SENP1-aP2KO mice (*n*=6, male) with an IgG isotype as a control. Bindings of p65/RelA to the IL-6, IL-1β and TNF-α gene promoters were quantified with the ratio of IP/input for each promoter (**j**). (**k**,**l**) Effects of SENP1 knockdown on NF-κB and cytokine expression in adipocytes. 3T3-L1 adipocytes were transfected with control or SENP1-specific siRNA for 24 h. IKK-NF-κB p65/RelA signalling molecules were detected by western blotting. A representative blot from three experiments is shown and protein levels are quantified by taking Ctrl as 1.0 (**k**). Protein levels of IL-6, TNF-α, IFN-γ and IL-1β levels present in adipocyte cultures were detected with ELISA after 24-h culture (**l**). All data are means±s.e.m. of three independent experiments. The two-tailed Student’s paired *t*-test was used for the statistical analysis. *P<0.05; **P<0.01, ***; *P*<0.001; DAPI, 4′,6-diamidino-2-phenylindole; IP, immunoprecipitation; NS, non-significance.

It has been proposed that adipose tissue depots originate from distinct precursor cells are functionally diverse, and modulate disease processes in a depot-specific manner^38^,^39^. We observed that peri-pancreatic adipocytes from SENP1-aP2KO exhibit 1.8-fold smaller cell sizes (Fig. 5a,b) with two- to three folds more cell numbers than the Ctrl adipocytes (Supplementary Fig. 5A–B). Moreover, peri-pancreatic adipocytes from SENP1-aP2KO mice expressed reduced differentiation markers including fatty acid synthase, adipose triglyceride lipase and lipoprotein lipase (Fig. 5c). In contrast, gonadal and subcutaneous inguinal adipocytes in SENP1-aP2KO mice exhibited a much weaker phenotype; they showed a smaller size at old ages (14 weeks) without significant increases in cell numbers; these phenotypes likely contribute to the reduced fat pad weight (Supplementary Fig. 5C–H) and total body weight of SENP1-aP2KO mice (Fig. 2). Similar results were obtained in SENP1-AdipoqKO and SENP1-PdgfRKO mice. These data are consistent with the notion that a reduced state of adipocyte differentiation correlates with enhanced proinflammatory phenotype in some adipose depots^38^,^39^,^40^.

Peroxisome proliferator-activated receptor-gamma (PPARγ) is not only the master regulator of adipogenesis but also plays a critical role in repressing inflammatory responses in a SUMOylation-dependent manner^12^,^41^. Therefore, we first examined whether PPARγ was a SENP1 target in adipocytes. We found that expression of total PPARγ mRNA and protein levels were similar in Ctrl and SENP1-deficient adipocytes (Supplementary Fig. 5I,J). Although PPARγ was SUMOylated in both Ctrl and SENP1-null adipocytes, no significant difference was detected between Ctrl and SENP1-deficient cells (Supplementary Fig. 5K,L). These results suggest that SUMOylation of PPARγ may not contribute to the reduced adipogenesis and enhanced cytokine production in PATs of SENP1-deficient mice.

NF-κB is a critical activator of genes for inflammation and immune regulation^42^. We observed that phosphorylations of IKK, IκBα and NF-κB subunit p65/RelA were increased with concomitant reduction of total IκBα in the adipose tissues of SENP1-aP2KO mice. In contrast, phosphorylation and total levels of ERK1/2 in adipose tissues were not affected by SENP1 deletion (Fig. 5d). NF-κB was further determined by intracellular staining for phosphor-p65/RelA in PAT sections. We observed phosphor-p65/RelA positive staining in the PAT, but not in the pancreas (Fig. 5e). The phosphor-p65/RelA was much stronger in SENP1-aP2KO adipocytes, marked by adipocyte-specific marker FABP4 (Fig. 5f,g). However, phosphor-p65/RelA was not localized with a macrophage marker F4/80 (Fig. 5h). We further performed p65/RelA chromatin immunoprecipitation (ChIP) assays in Ctrl and SENP1-deficient cells isolated from Ctrl and SENP1-aP2KO mice. We observed increased p65/RelA bindings to the IL-6, IL-1β and TNF-α promoters in SENP1-aP2KO adipocytes (Fig. 5i,j). To directly test whether SENP1 deficiency regulates the NF-κB activation and cytokine production in adipocytes *in vitro*, 3T3-L1 preadipocytes were first differentiated into the mature adipocytes and then transfected with a Ctrl or SENP1 short interfering RNA (siRNA). In the adipocytes with the SENP1 knockdown, phosphorylated IKK, IκBα and NF-κB p65/RelA levels were increased markedly (Fig. 5k). The gene expressions of proinflammatory cytokines (IL-6, TNF-α and IL-1 β, but not IFN-γ) from adipocytes were significantly higher in SENP1 knockdown cells (Fig. 5l), which concurs with our observations in SENP1-AdipoqKO and SENP1-aP2KO mice, as well as in adipocytes isolated from these mice. Conversely, overexpression of SENP1 into SENP1-deficient adipocytes markedly decreased phospho-p65/RelA and cytokine productions. Taken together, our results suggest that SENP1 directly regulates the activation of NF-κB and cytokine production in adipocytes.

### SENP1 deletion augments NEMO SUMOylation in adipocytes

**Figure 6:**
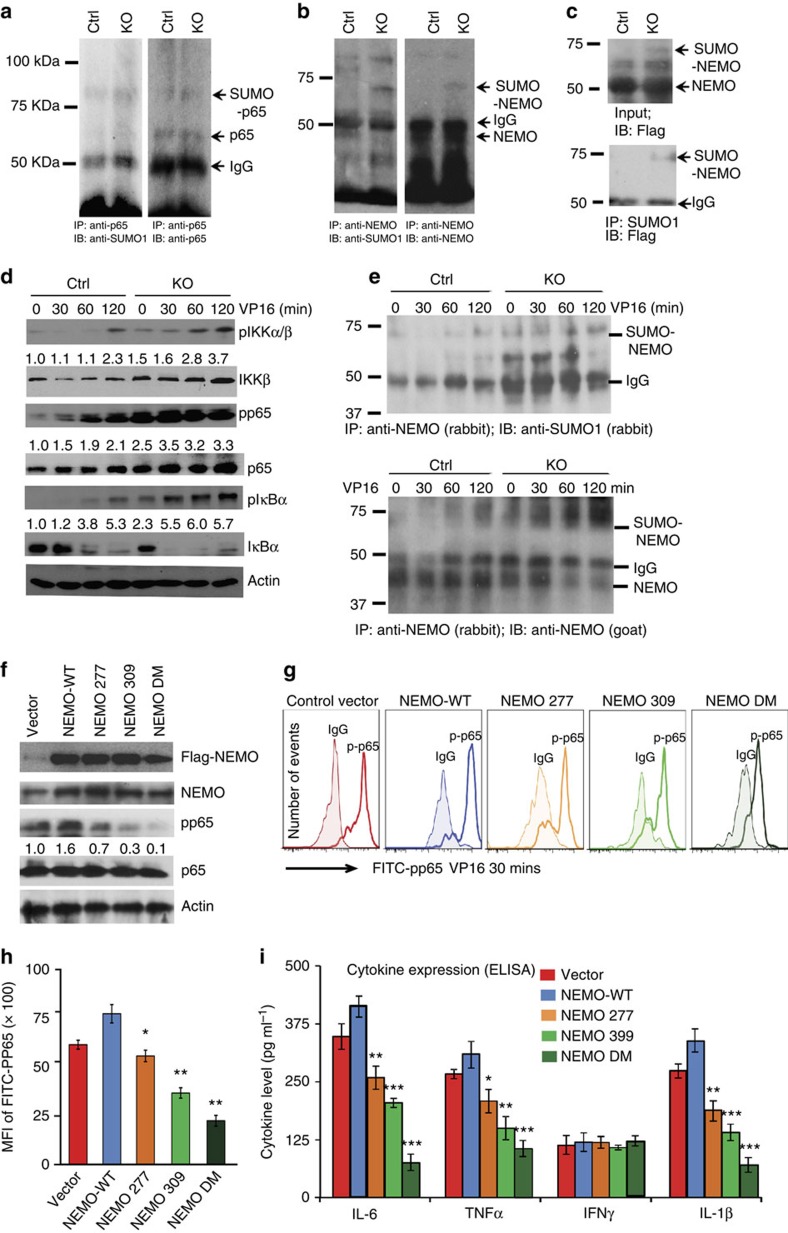
SENP1 deletion augments NEMO SUMOylation and cytokine expression in the adipocytes. (**a**,**b**). SUMOylation of NEMO, but not p65/RelA, was enhanced in the adipocytes of SENP1-aP2KO mice. Proteins extracted from the adipocytes of Ctrl and SENP1-aP2KO mice at the age of 5 weeks were subjected to immunoprecipitation with p65/RelA (**a**) or NEMO (**b**) antibodies followed by western blotting with anti-SUMO1, anti-p65/RelA or anti-NEMO. Proteins are indicated. Representative blots from one pair of Ctrl and SENP1-aP2KO mice are shown. Similar results were obtained from additional two pairs of mice. (**c**) Flag-tagged NEMO was transfected into adipocytes from Ctrl and SENP1-aP2KO mice at the age of 5 weeks. Proteins extracted were subjected to immunoprecipitation with SUMO1 antibody followed by western blotting with anti-flag. Input for flag-NEMO was detected with flag antibody. Representative blots from one pair of Ctrl and SENP1-aP2KO mice are shown. Similar results were obtained from additional two pairs of mice. (**d**) Effect of SENP1 deletion on stress-induced IKK activation, p65/RelA phosphorylation and NEMO SUMOylation. Adipocytes from Ctrl and SENP1-aP2KO mice were treated with VP16 (10 μM) for indicated times. IKK-NF-κB p65/RelA signalling molecules were determined by western blot. Ratios of p-IKK/IKK, p-p65/RelA/p65/RelA and pIκB-α were quantified by taking Ctrl as 1.0. Representative blots from one pair of Ctrl and SENP1-aP2KO mice are shown. Similar results were obtained from additional two pairs of mice. (**e**) VP16-induced NEMO SUMOylation was determined by co-immunoprecipitation assay with anti-NEMO (a rabbit polyclonal IgG) followed by immunoblotting with anti-SUMO1 (a rabbit polyclonal) and anti-NEMO (a goat polyclonal IgG). Representative blots from three independent experiments are shown. (**f**) Primary adipocytes isolated from the adipose tissue of SENP1-aP2KO mice at the age of 5 weeks were reconstituted with Flag-tagged NEMO-WT, K277R, K309R or K277/309R (DM) as detected by immunoblotting. Phosphor-p65 was quantified by taking Ctrl as 1.0. (**g**,**h**) Effect of NEMO mutants on phosphor-p65 in adipocyte was detected by intracellular staining with anti-p-p65 and FITC-conjugated secondary antibody followed by FACS with isotype IgG as a control. Representative FACS results are shown in **g** with quantifications in **h** from three independent experiments. (**i**) Effect of NEMO mutants on cytokine expression. Protein levels of IL-6, TNF-α, IFN-γ and IL-1β levels present in adipocyte cultures were detected with ELISA after 24-h culture. All data are means±s.e.m. of three independent experiments. The two-tailed Student’s paired *t*-test was used for the statistical analysis. *P<0.05; **P<0.01; ***P<0.001; Ctrl, control; IB, immunoblotting; IP, immunoprecipitation; KO, knockout.

Of the NF-κB components, SUMOylation of IKK subunit NEMO and NF-κB p65/RelA are thought to be critical events for the activation of IKK-NF-κB signalling^9^,^10^,^43^,^44^. SENP2 can efficiently deSUMOylate NEMO to blunt NF-κB activation in a 293T overexpression system^45^ and SENP1 was proposed to have a similar role^46^. To determine the role of SENP1 in pancreatic proinflammatory phenotype and NF-κB regulation, we first examined expression of SENPs in PATs. The SENPs can be divided into three families. The first family members SENP1 and SENP2 have broad specificity for the three mammalian SUMOs (SUMO1–3), while the second family (SENP3 and SENP5) and the third family (SENP6 and SENP7) prefer SUMO2/3 as substrates^14^. We found that SENP1, but not SENP2, was abundantly expressed in mouse adipose tissues. Deletion of SENP1 had no effects on expression of other SENPs (Supplementary Fig. 6). SUMOylations of NEMO and p65/RelA were determined by co-immunoprecipitation from the denaturing adipocyte extracts with specific antibodies followed by western blotting with anti-SUMO1. No difference in p65/RelA was detected between Ctrl and SENP1-aP2KO mice (Fig. 6a). However, an extra band appeared ∼70 kDa in the SENP1-aP2KO adipocyte extracts for NEMO (Fig. 6b). Increased NEMO SUMOylation in SENP1-KO adipocyte was further confirmed by overexpression of Flag-NEMO (Fig. 6c). It is reported that NEMO SUMOylation and IKK activation are enhanced by stress responses such as oxidative stress, inflammatory stimuli and DNA damage in different cell types^9^,^47^. We observed that DNA damage agent etoposide VP16 induced phosphorylation of IKK and p65/RelA in adipocytes, and SENP1 deletion enhanced both basal and stress-induced IKK and p65/RelA activation (Fig. 6d). SENP1 deletion also enhanced both basal and stress-induced NEMO SUMOylation in adipocytes as detected by co-immunoprecipitation with anti-NEMO followed by immunoblotting with anti-SUMO1 (Fig. 6e).

It has been reported that NEMO can be SUMOylated at K277 and K309 (ref. 9). To determine the functional importance of SUMO modifications in regulating NF-κB signalling in adipocytes, a single (K277R or K309R) and double lysine to arginine mutants (DM) were constructed as Flag-NEMO. Wild type (WT) or mutant Flag-NEMO was then was expressed in peri-pancreatic adipocytes isolated from SENP1-aP2KO mice, and the exogenous NEMO versus endogenous NEMO was about 1:1 ratio (Fig. 6f). While phosphorylation of NF-κB p65/RelA was slightly increased by NEMO-WT, it was mildly decreased by NEMO K277R and strongly decreased by NEMO K309R or the double mutant as detected by immunoblotting (Fig. 6f) and intracellular staining with fluorescein isothiocyanate (FITC)-conjugated p-p65/RelA (Fig. 6g,h). These data suggest that SUMO-deficient NEMO mutants (NEMO K309R and NEMO-DM) function as dominant negative forms. NF-κB-dependent cytokine expression was then determined by ELISA in the NEMO-transfected cells. NEMO mutants had similar effects on the protein expression of proinflammatory cytokines in adipocytes (Fig. 6i), supporting that SUMOylated NEMO enhances cytokine expression.

### Clinical relevance of adipocyte-specific SENP1-deficient mice

**Figure 7:**
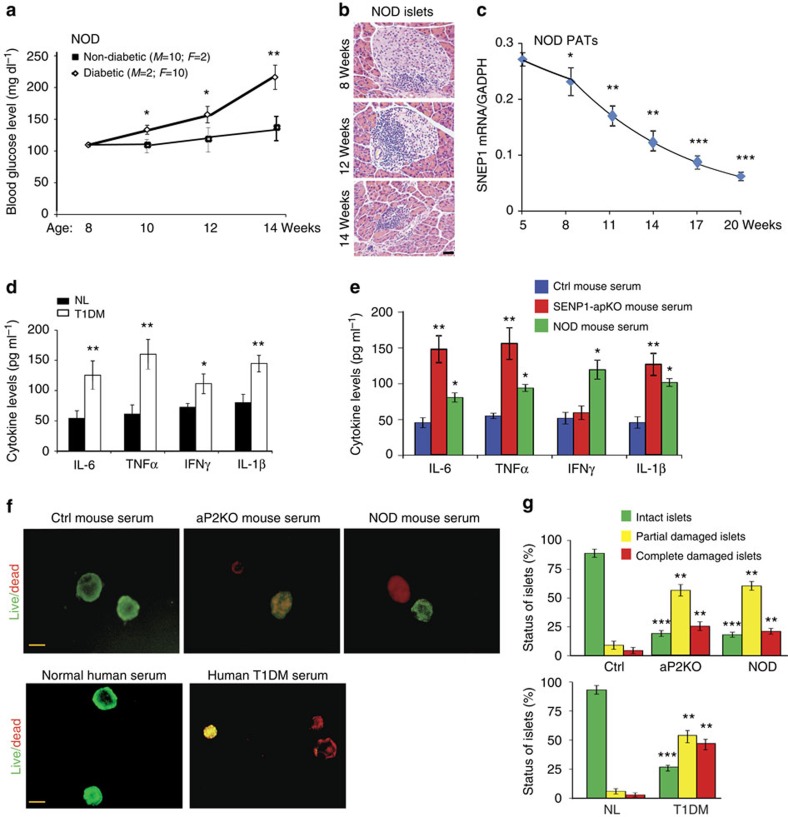
Clinical relevance of adipocyte-specific SENP1-deficient mice as a diabetic model. (**a**–**c**) Diabetic phenotype in NOD mice. Tweelve pairs of age-matched male and female NOD mice were performed for diabetic assays at various ages. (**a**) Glucose levels at the ages of 8, 10, 12 and 14 weeks were measured. (**b**) Haematoxylin and eosin stain staining for overall morphology of the pancreases. Representative images of the pancreases for the age of 8, 12 and 14 weeks are shown. Three sections from each islet and *n*=6 mice per group at each age were examined. Scale bar, 20 μm. (**c**) Correlation of SENP1 expression in adipocytes with T1DM development in NOD mice from the ages of 5–20 weeks. SENP1 expression in the PATs of NOD mice at various ages was detected by quantitativePCR with reverse transcription. (**d**–**g**) Direct effect of sera from SENP1-aP2KO, NOD and human T1DM on islets. (**d**) Proinflammatory cytokines, TNF-α, IL-1β, IL-6, as well as IFN-γ from human plasma were measured by ELISA. Data are means±s.e.m. from age-matched Ctrl (*n*=21) and T1DM patients (*n*=29). (**e**) Proinflammatory cytokines,TNF-α, IL-1β and IL-6 and IFN-γ in the serum of Ctrl, SENP1-aP2KO and NOD mice at the age of 12 weeks were measured by ELISA, *n*=6, male. (**f**) Mice pancreatic islets (100 islets per well) were cultured with a serum for 72-h incubation. Islet structure and apoptosis were monitored by Live/Dead viability/Cytotoxicity kit and detected by fluorescence microscopy. Representative images from each group are shown. Scale bar, 200 μm. (**g**) Quantifications of intact islets (green), partial damaged islets (yellow) and completed damaged islets (red). Total 300 islets from each group were counted. Serum from SENP1-aP2KO mice, NOD mice and T1DM patients all significantly induced islet damages compared with Ctrl mouse serum. Data are means±s.e.m. of three independent experiments. The two-tailed Student’s paired *t*-test was used for the statistical analysis, *P<0.05; **P<0.01; ***P<0.001; Ctrl, control.

To determine whether our studies from the adipocyte-specific SENP1-deficient mice have clinical relevance, we first investigated whether there is a correlation between SENP1 expression in adipocytes of non-obese diabetic (NOD) mice and human T1DM patients. The NOD mouse, a popularly used model for T1DM, developed diabetes with a late onset at the age of 14 weeks^48^. Consistent with reports, we observed the incidence of spontaneous diabetes in the NOD mouse was 60–80% in females and 20–30% in males (Fig. 7a). Mouse strains with an adipocyte-specific deletion of SENP1 appeared more severe in hyperglycaemia and islet disruption compared with NOD mice at the same age (Fig. 7b versus Fig. 3a; Supplementary Fig. 2). Importantly, we observed an age-dependent reduction of SENP1 expression in PATs of NOD mice (Fig. 7c), correlating with the T1DM progression in NOD mice.

We next investigated a correlation between SENP1 expression and proinflammatory cytokines in NOD model and human T1DM patients. To this end, venous blood was drawn from consenting volunteers (healthy and diabetic subjects; Supplementary Table 1). Plasma levels of proinflammatory cytokines were significantly increased in T1DM patients compared with the normal cohorts (Fig. 7d). The levels of cytokines from SENP1-aP2KO at the age of 12 weeks are at similar ranges as human serum with T1DM. NOD develops diabetes as a result of Th1 dominant IFN-γ-induced insulitis and leukocyte infiltrations of the pancreatic islets^48^. Consistently, NOD mice exhibit increased plasma IFN-γ with lower levels of proinflammatory cytokines compared with SENP1-aP2KO mice and serum from human T1DM patients (Fig. 7e versus Fig. 7d). We then tested whether serum could directly induce β-cell death and pancreatic disruption. To this end, isolated normal mouse pancreatic islets were cultured with serum collected from Ctrl, SENP1-aP2KO and NOD mice, or collected from normal human and T1DM patients. Islet structure and apoptosis was monitored by Live/Dead viability/Cytotoxicity assays. Serum from SENP1-aP2KO mice, NOD mice and human T1DM all induced disruption and pancreatic cell death (Fig. 7f,g). Taken together, our results support that SENP1-aP2KO mice is a clinically relevant model for human diabetes.

We also tested whether insulin could prevent or delay T1DM progression in SENP1-aP2KO mice. Since T1DM is an islet-reactive T-cell-mediated autoimmune disease and insulin and its precursor (pre)proinsulin are major targets of islet-reactive T cells, both in humans and in NOD mice^49^. It has been hypothesized that that repeated subcutaneous insulin administration prevents disease by acting as a vaccination protocol potentially restoring immune tolerance^50^. Recent data suggest that short-term subcutaneous insulin administration delays but does not prevent diabetes in NOD mice, likely by inducing a state of ‘β-cell rest’ that would make β cells less vulnerable to metabolic stress, apoptosis and possibly to immune-mediated destruction^49^,^51^. Our results indicated that prophylactic insulin therapy in SENP1-deficient mice at the age of 5 weeks for 9 weeks only partially ameliorates development of diabetes phenotypes including immune cell infiltration, β-cell apoptosis and islet disruptions (Supplementary Fig. 7A–F).

### NF-κB inhibitors ameliorate diabetes progression

**Figure 8:**
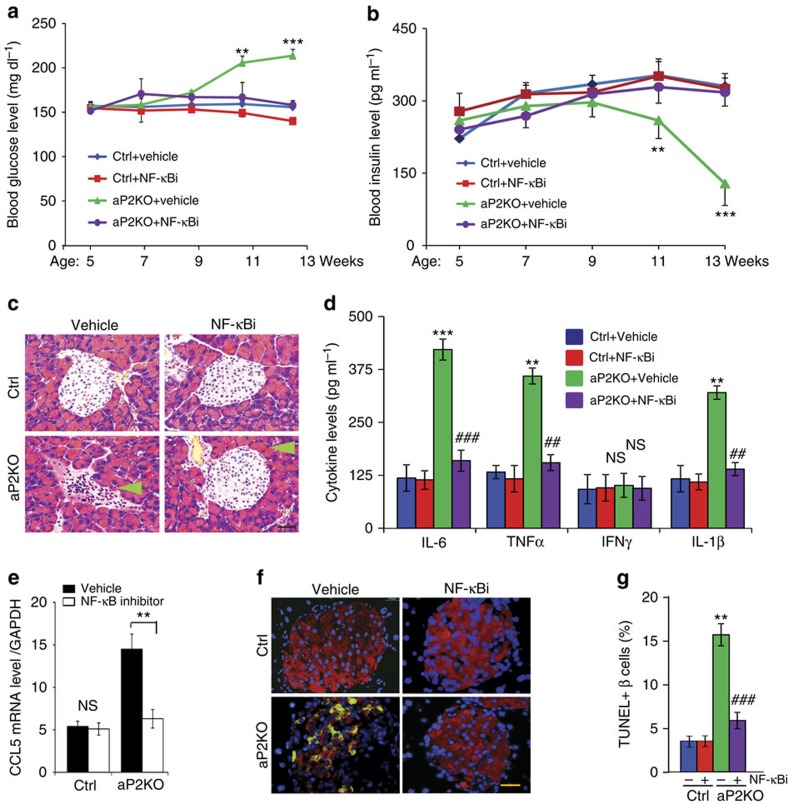
Inhibition of NF-κB activity prevents T1DM progression in SENP1-deficient mice. (**a**–**g**) NF-κB inhibitor rescued the diabetic phenotype. Ten pairs of age-matched Ctrl and SENP1-aP2KO male mice were treated with NF-κB Activation Inhibitor II, JSH-23 or vehicle at 2 mg kg^−1^ body weight twice a week from the age of 5 weeks for 6 weeks. (**a**,**b**) Glucose and insulin levels in Ctrl vehicle, Ctrl JSH-23, SENP1-aP2KO vehicle and SENP1-aP2KO JSH-23 mice at the ages of 5, 7, 9, 11 and 13 weeks were performed. (**c**) Tissues were collected at the age of week 13. The overall morphology of the pancreases was compared using H&E staining. Representative images of the pancreases from 10 pair mice are shown. Arrowheads indicate structural changes in the islets of SENP1-aP2KO mice with or without JSH-23. (**d**) Cytokines IL-6, TNF-α, IFN-γ and IL-1β proteins from PATs were measured by ELISArray kits. (**e**,**f**) CCL5 level in the islet of Ctrl vehicle, Ctrl JSH-23, SENP1-aP2KO vehicle and SENP1-aP2KO JSH-23 mice at the age of 13 weeks were measured by quantitativePCR with reverse transcription with GAPDH for normalization (**e**) and immunostaining with anti-CCL5 (**f**). (**g**) β-Cell apoptosis. TUNEL *in situ* cell death staining together with β-cell marker insulin was performed in pancreas. TUNEL-positive β cells are quantified. All results are shown as means±s.e.m., *n*=10 male mice in each group. The two-tailed Student’s paired *t*-test was used for the statistical analysis, *P<0.05; **P<0.01; ***P<0.001, compared with Ctrl group; #*P*<0.05; ##*P*<0.01; ###*P*<0.001, compared with KO group with versus without JSH-23. Ctrl, control; NS, non-significance.

To establish a causative link between SENP1-dependent NF-κB activation and type 1 diabetes, SENP1-aP2KO mice were treated at the age of 5 weeks with intraperitoneal injection of NF-κB activation inhibitor II JSH-23 (2 mg kg^−1^ in 10% dimethylsulphoxide (DMSO) diluted with PBS) twice a week for 6 weeks. JSH-23 had no effects on body weight, blood glucose and lipid profile in the Ctrl mice. However, administration of JSH-23 in SENP1-aP2KO mice diminished SENP1 deletion-induced elevation of blood glucose (Fig. 8a) and reduction of insulin levels in blood (Fig. 8b) concomitant with attenuated islet damages (Fig. 8c). These normalized phenotypes in JSH-23-treated SENP1-aP2KO mice correlated with basal levels of proinflammatory cytokines in the PATs of these mice (Fig. 8d). No CCL5 expression in islets (Fig. 8e,f) and apoptotic β-cells (Fig. 8g) were detected in the JSH-23-treated group. These results support a critical role of SENP1-dependent NF-κB activity in T1DM progression in SENP1-aP2KO mice.

### HFD augments diabetic phenotype in SENP1 deficient mice

**Figure 9:**
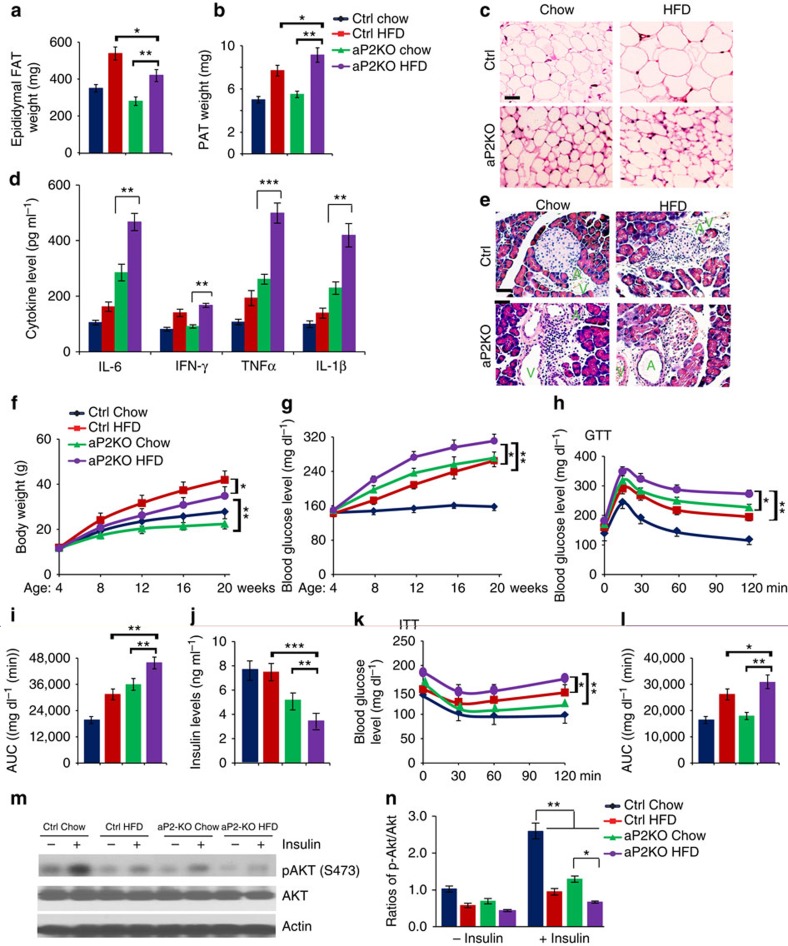
HFD augments diabetic phenotype in adipocyte-specific SENP1-deficient mice. Ctrl and SENP1-aP2 KO male mice were fed at the age of 4 weeks with normal chow (Chow) or high-fat diet (HFD) for 4–16 weeks. (**a**–**c**) Effect of HFD on adipose tissues. Epididymal white adipose tissue (**a**) and PATs (**b**) were collected and weighed at the age of 12 weeks. PAT morphology of PAT (**c**) was visualized by H&E staining. *n*=6 male mice per group. (**d**) Cytokine levels in PATs were measured at the age of 8 weeks. *n*=6 male mice for each group. Scale bar, 20 μm. The two-tailed Student’s paired *t*-test was used for the statistical analysis. Data are presented as means±s.e.m. *P<0.05; **P<0.01, ***P< 0.001. (**e**) Overall morphology of pancreas was visualized by H&E staining. Representative images of the pancreases from mice at the age of 12 weeks are shown. Three sections from each mice, *n*=6 male mice per group. Scale bar, 20 μm. (**f**,**g**) Body weights (**f**) and glucose levels (**g**) were measured at the indicated ages, *n*=12 male mice in each group. ANOVA *P<0.05; **P<0.01. (**h**–**j**) GTT assay at the age of 12 weeks. Mice were fasted overnight, followed by an intraperitoneal injection of glucose (1 g kg^−1^ body weight). Blood glucose levels at 0–120 min were measured (**h**) and AUC at 120 min was calculated. *n*=10, male for each group. ANOVA, *P<0.05; **P<0.01. (**i**). Serum insulin levels at 15 min during GTT were measured. ANOVA, *P<0.05; **P<0.01 (**j**). (**k**,**l**) ITT assay at the age of 12 weeks. Mice were fasted overnight, followed by an intraperitoneal injection of insulin (0.75 g kg^−1^ body weight). Blood glucose levels were determined by an electronic glucometer (**k**) and AUC was calculated (**l**). *n*=10 male for each group. ANOVA, *P<0.05; **P<0.01. (**m**,**l**) Insulin responses at the age of 12 weeks. Mice were injected (intraperitoneally 10 U kg^−1^ body weight for 8 min). Akt expression and phosphorylation level in the liver were detected by western blotting. A representative blot from *n*=4 per group is shown (**m**). Normalized ratios of p-Akt/Akt were quantified in (**n**) by taking Ctrl chow as 1.0. *n*=4. The two-tailed Student’s paired *t*-test was used for the statistical analysis. Data are presented as means±s.e.m. *P<0.05; **P<0.01, ***P< 0.001. Designated colour bars indicate four groups of mice: Ctrl Chow, dark blue; Ctrl HFD, red; aP2KO Chow, green; aP2KO HFD, purple; ANOVA, Analysis of variance; AUC, area under curve; Ctrl, control, wks, weeks.

To determine the role of the SENP1-NEMO-NF-κB-pathway in adipocyte inflammation and its link to both T1DM and T2DM, we have examined the phenotype of SENP1-aP2KO mice under high-fat diet (HFD) conditions. HFD increased fat weight in all adipose depots including epididymal fat and PATs in Ctrl and SENP1-aP2KO mice (Fig. 9a,b). PATs from SENP1-aP2KO exhibit 1.8-fold smaller cell sizes with 2–3-fold more cell numbers than the Ctrl adipocytes under normal chow and this phenotype was more marked under HFD (Fig. 9c), suggesting that HFD may stimulate adipogenesis in PATs of SENP1-aP2KO. PATs from SENP1-aP2KO mice expressed high levels of IL-6, TNF-α and IL-1β at the age of 7 weeks before the onset of diabetes, and these proinflammatory cytokines were higher in the PATs compared with other adipose depots (Fig. 4). HFD significantly augmented the cytokine production in PATs (Fig. 9d) and subsequent disruption of islet structures in SENP1-aP2KO (Fig. 9e). Consistently, HFD increased body weight (Fig. 9f) and accelerated hyperglycaemia with an onset at the age of 8 weeks (versus 12 weeks under chow) in SENP1-aP2KO mice (Fig. 9g). Basal and glucose-stimulated insulin levels were significantly reduced in SENP1-aP2KO mice. Moreover, HFD caused further reduction of insulin levels in SENP1-aP2KO mice, but not in Ctrl mice (Fig. 9h–j). Although similar insulin responses were observed between SENP1-deficient mice and Ctrl under standard chow in the ITT assay, HFD significantly induced insulin resistance in Ctrl mice and more severe phenotype was observed in SENP1-aP2KO mice (Fig. 9k,l). Moreover, liver insulin resistance, a hallmark of obesity-linked T2DM development, was detected under basal but more pronounced under HFD conditions in SENP1 knockout (KO) mice (Fig. 9m,n). Taken together, our data demonstrate that HFD augments both T1DM and T2DM phenotypes in SENP1-aP2KO mice.

## Discussion

**Figure 10:**
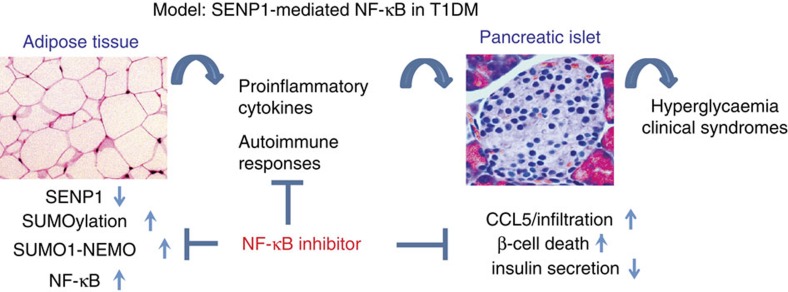
A model for the role of SENP1 in T1DM. SENP1 keeps NF-κB and inflammation in quiescent in adipose tissues. Here we show that adipocyte-specific SENP1 deletion induces NEMO SUMOylation, NF-κB activation and NF-κB-dependent proinflammatory cytokine production in adipose tissues, profoundly in the peri-pancreatic adipose tissue (PAT). These cytokines induce high levels of chemokine CCL5 expression in adjacent islets to recruit CCR5^+^ immune cells. Subsequently, the cytokines and activated immune cells, especially CD8^+^ and CD4^+^T cells, attack the pancreases, leading to the chronic destruction of the islet structures, damaged β cells, autoantibody onset and type-1 diabetes progression in the SENP1-deficient mice. Therefore, NF-κB inhibitors block inflammation and ameliorate diabetes progression in the SENP1-deficient mice. SENP1 expression in PATs of the diabetic NOD mice is reduced in an age-dependent manner, correlating with the diabetic progression in these mice. Our current study demonstrates that reduced SENP1 expression and enhanced NF-κB activity in PATs may represent a common mechanism for the role of protein SUMOylayion in the pathogenesis of T1DM.

In the present study, we have employed three different adipocyte-specific Cre deleter lines (Adipoq-Cre, PdgfRα-Cre and aP2/FABP4-Cre) and demonstrated that adipocyte-specific SENP1-deficient mice gradually develop a T1DM phenotype, including hyperglycaemia, reduced insulin secretion, glucose intolerance and weight loss. The adipocyte-specific SENP1 deletion induces NF-κB activation and NF-κB-dependent proinflammatory cytokine production in the PATs. These cytokines induce high levels of CCL5 expression in adjacent islets to recruit CCR5^+^ immune cells. Subsequently, the cytokines and activated immune cells, especially CD8^+^ and CD4^+^T cells, attack the pancreases, leading to the chronic destruction of the islet structures, damaged β cells, autoantibody onset and diabetes progression in the SENP1-deficient mice (Fig. 10). Local and circulating proinflammatory cytokines may serve as a mechanism to break self-tolerance and augment cytotoxic immune response in pancreases islets, contributing to the onset of T1DM. The NOD mouse is a popularly used model for T1DM and develops diabetes as a result of Th1 dominant IFN-γ-induced insulitis and leukocytic infiltrations of the pancreatic islets^48^. Therefore, Th1 and IFN-γ play the key role in the disease onset in NOD mice, which can capture some characteristics of human T1DM, but not the whole disease progression processes. Our adipocyte-specific SENP1-deficient mice recapitulate the evolution process of T1DM from preclinical stage to later phase of T1DM, and therefore may provide a unique model for human T1DM. Unlike NOD mice, both male and female SENP1-deficient mice exhibit similar diabetic phenotype. It is worthy to mention that adipocyte-specific SENP1-deficient mice exhibit normal lipid profiles in total cholesterol, low-density lipoprotein, HDL, TG and FFA upto the age of 14 weeks, suggesting that inflammation, but not lipotoxicity^52^, induced β-cell damages contribute to diabetic phenotype in SENP1-aP2KO mice. Of note, peri-pancreatic adipocytes from SENP1-deficient mice exhibit reduced differentiation markers with a heightened proinflammatory state compared with other tissues, supporting the profound diabetic phenotype in these mice. Mechanistic studies suggest that SENP1 deletion in adipocytes causes NF-κB activation and NF-κB-dependent cytokine expression. The high SUMOylation of the NF-κB component NEMO at lysines-277/309 is the key factor in inducing NF-κB activation and cytokine expression in SENP1-deficient adipocytes. Importantly, NF-κB inhibitors ameliorate diabetes progression. Our data support that SENP1-mediated NEMO deSUMOylation in the peri-pancreatic adipocytes limits inflammatory responses and diabetes progression. It is conceivable that NEMO SUMOylation and NF-κB activation in PATs is more susceptible to alterations of SENP1 expression/activity versus other adipose depots. Alternatively, pancreas and pancreatic β-cells are more sensitive to local and systemic cytokine-mediated immune responses.

It has been reported that aP2-Cre deletion is floxed gene loci specific^23^. The majority of studies have reported highly adipocyte-specific recombination of the *aP2/Fabp4* promoter-driven Cre strain (aP2-Cre^BI^)^24^,^25^,^26^,^53^. A couple of reports have shown variable degrees of target gene recombination in macrophages^27^,^54^. A recent study suggested that the aP2-Cre induces recombination in the capillary endothelium in the heart and in intermyofibrillar cells in the skeletal muscle, but not in macrophages in adipose tissue^23^. Interestingly, they have found that different floxed gene loci display differential sensitivity to Cre-mediated recombination and that different adipose depots recombine to different extents. Therefore, it is necessary that one should carefully examine the specific gene deletion in their study. We did not detect significant differences in SENP1 expression in brain, muscle and liver tissues between WT and SENP1-aP2KO mice. Therefore, the deletion of SENP1 was limited to the adipose tissues in the SENP1-aP2KO mice. Our results are consistent with a recent report^55^. With the same aP2-Cre strain as in our study, the authors have shown that aP2-cre expression is highly specific for adipocytes, and they found no Cre-mediated target gene recombination in muscle, liver, brain, adipose stromal vascular cells or adipose macrophages.

A novel mechanistic insight from our study is the ability of SENP1 to control NF-κB-dependent proinflammatory cytokine secretion in adipocytes. It has been shown that only SENP2, but not other SENPs, can efficiently deSUMOylate NEMO and enhance NF-κB activation induced by DNA damage in a HEK 293T overexpression system^45^. Our data indicate that SENP1 expression is sixfold higher than SENP2 in mouse adipose tissues. Moreover, our studies using siRNA knockdown, SENP1-KO adipocytes and reconstitutions of SENP1 into SENP1-KO cells clearly demonstrate that SENP1, by deSUMOylating NEMO at K277 and K309, plays a pivotal role in regulating NF-κB activation and cytokine production in adipocytes. Given that SENP1 and SENP2 are two members of SENPs with broad specificity for SUMOs^14^,^15^, it is conceivable that SENP1 and SENP2 regulate NEMO SUMOylation and NF-κB activation in different tissues and/or cell types. Our data is in line with the report that components in SUMOylation are associated with T1DM susceptibility. Interestingly, a variant of SUMO4 (M55V) strongly associated with T1DM results in 5.5 times greater NF-κB transcriptional activity by conjugating to NF-κB inhibitor IκBα^18^,^19^. Although a genetic mutation of SENP1 has not been identified thus far, our study suggests that reduced SENP1 expression and enhanced NF-κB activity in adipose tissues may represent a common mechanism for the role of SUMOylayion in the pathogenesis of T1DM. Of clinical significance of our current work, we observe an age-dependent reduction of SENP1 expression in PATs of the diabetic NOD mice, correlating with the diabetic progression in these mice.

Typical features of human T1DM include hyperglycaemia, cytotoxic effector cell-mediated attack on β cell, β-cell death and generation of autoantibodies^1^,^2^,^3^. These phenotypes are evidently observed in the adipocyte-specific SENP1-deficient mice, suggesting that SENP1 in adipocytes plays a pivotal role in preventing autoimmune diabetes. Although previous research focuses on lipid metabolism in the adipocytes, it is now recognized that adipose tissue is the largest endocrine organ that could play an important role in the production of proinflammatory cytokines, such as IL-1β, IL-6 and TNF-α, to regulate immune responses^5^,^56^. Our data demonstrate that T1DM onset in the SENP1-deficient mice is strongly associated with high levels of proinflammatory cytokines secreted by PATs, underlying a mechanistic link between peri-pancreatic adipocytes and diabetes. First, proinflammatory cytokines have direct effects on islet immunogenicity and islet β-cell survival. We show that the high concentrations of PAT-derived cytokines directly induce expression of CCL5 and other chemokines (such as CCL2, CCL21, CXCL9 and CXCL10) in adjacent pancreatic islets, chemokines responsible for recruitment of immune cells. It needs to further investigate if and how CCL5 and other chemokines directly contribute to T1DM progression. The inflammatory cytokines have direct cytotoxic effects on islet β cells. Second, proinflammatory cytokines have direct effects on various immune cell types, specifically T cells. These immune cells further release more innate inflammatory cytokines, which damage β cells and break tolerance through activation of adaptive responses. Indeed, we observe that Th1 and Th17 effector T-cell subsets expanded in SENP1-deficient mice. Third, proinflammatory cytokines and cytotoxic T cells triggers β-cell turnover^2^,^57^, resulting in the release of autoantigens and endogenous ‘danger signals’ capable of promoting pathologic self-antigen presentation^58^. We observe massive apoptosis of β cells in our mouse model, accompanying with infiltrations of CD8^+^ and CD4^+^ T cells. Indeed, we detect IAA in the SENP1-aP2KO mice at old ages, indicative of autoimmune disease T1DM in these mice. A reduced number of Foxp3^+^ regulatory T cells are also observed in SENP1-deficient mice. Moreover, islet-specific autoimmune NRP-V7-positive CD8^+^ T cells could be detected in the pancreatic lymph nodes and the spleen of SENP1-deficient mice. Taken together, our study provides a strong mechanistic link between adipose-secreted proinflammatory cytokines and T1DM. It will be important to test whether blockade of proinflammatory cytokines or blockade of cytotoxic T cells could block T1DM pathogenesis development in the SENP1-deficient mouse model.

It worth mentioning that type-1 and type-2 diabetes are systemically linked. Specifically, the accumulation of activated innate immune cells in metabolic tissues results in release of inflammatory mediators, in particular, IL-1β and TNF-α, which promote systemic insulin resistance and β-cell damage^6^. Our data demonstrate that SENP1-aP2KO mice have strong T1DM phenotypes concomitant with mild T2DM, which can be exacerbated by HFD. Specifically, HFD augments PAT morphological change, cytokine production and pancreatic damage in SENP1-aP2KO mice, and HFD augments hyperglycaemia and insulin reduction in SENP1-aP2KO mice. Moreover, a moderate insulin resistance in the liver of SENP1-deficient mice is observed under standard chow. However, HFD augments liver insulin resistance, a hallmark of obesity-linked T2DM development, in SENP1-aP2KO mice. Taken together, our data demonstrate that the increased cytokines in SENP1-aP2KO mice contribute not only to T1DM progression but also to HFD-induced T2DM phenotypes in these mice.

In summary, our experiments define the role of the SENP1-mediated protein SUMOylation in pancreatic immune responses, β-cell damages and consequently diabetes progression. Our study has provided a molecular mechanism for the pathogenesis of diabetes, and may help to develop a novel therapeutic strategy for treatment of diabetes.

## Methods

### Adipocyte-specific SENP1 knockout mice

SENP1^+/lox^ mice were generated by inserting loxP sites surrounding the *SENP1* gene exons 5 and 6, based on homologous recombination^17^. SENP1^lox/lox^ mice were obtained by intercrossing SENP1^+/lox^ mice. SENP1^lox/lox^ mice were mated with three different deleter lines carrying the Cre recombinase driven by the *Adiponectin* (obtained from Jackson Laboratory), *PdgfR*α (obtained from Dr Matthew Rodeheffer at Yale University School of Medicine) or *aP2/Fabp4* gene promoter (BI line from Dr Barbara Kahn, Beth Israel Deaconess Medical School, Harvard Medical School). All mice had been subsequently backcrossed onto the C57BL/6 background for >6th generations. The deletion of *SENP1* in adipocytes of SENP1^lox/lox^:Cre was verified by quantitativePCR with reverse transcription using primers amplifying exons 5–6 (ref. 17) and specific Cre. SENP1^+/+^:Cre and SENP1^lox/lox^ mice used as controls. Mice were cared for in accordance with National Institutes of Health guidelines, and all procedures were approved by the Yale University Animal Care and Use Committee.

### HFD challenge

Mice were fed a standard chow containing 10% fat, 70% carbohydrate 20% protein (D12450B, OpenSource DIETS) or a HFD containing 45.0% fat, 35.0% carbohydrate and 20% protein (D12451; OpenSource DIETS, New Brunswick, NJ, USA) at 4 weeks of age for 4–16 weeks. All experiments were approved by the Institutional Animal Care and Use Committee (IACUC) of Yale University.

### Rescue of diabetic phenotype by NF-κB inhibitor

NF-κB Activation Inhibitor II JSH-23 is an inhibitor of NF-κB transcriptional activity (half-maximal inhibitory concentration=7.1 μM) by inhibiting the mechanism of transcription, without affecting the process of IκBα degradation. To address whether the NF-κB activation inhibitor rescued diabetic phenotype in mice, SENP1^lox /lox^ and SENP1^lox /lox^: aP2-Cre were intraperitoneally injected with NF-κB Activation Inhibitor II (JSH-23; sc-222061; at 2 mg kg^−1^ in 10% DMSO diluted in PBS) twice per week from the age of 5 weeks for 6 weeks. Vehicle (10% DMSO in PBS) was used as a control. Tissues were collected at week 13.

### Glucose-stimulated insulin secretion

Experiments were performed in static incubation^59^. In brief, islets (100 islets per well) were preincubated for 30 min at 37 °C in Kreb’s buffer (140 mmol l^−1^ NaCl, 30 mmol l^−1^ HEPES, 4.6 mmol l^−1^ KCl, 1 mmol l^−1^ MgSO_4_, 0.15 mmol l^−1^ Na_2_HPO_4_, 5 mmol l^−1^ NaHCO_3_, 2 mmol l^−1^ CaCl_2_ and 0.05% bovine serum albumin (BSA), pH 7.4) containing 3 mmol l^−1^ D-glucose. Then, the plates were cooled down on ice water and the supernatants were replaced with cold Kreb’s buffer containing 16.7 mmol l^−1^ D-glucose. After a 30-min incubation at 37 °C, the insulin released into the supernatant was measured. Concentrations of insulin (ng ml^−1^) in the supernatants were directly used to evaluate insulin secretion.

### Metabolic characterization

The control or SENP1-aP2KO mice were fed with normal chow, water consumption and food intake was recorded daily. For the GTT and ITT, the mice were fasted overnight, followed by an intraperitoneal injection of glucose (GTT; 1 g kg^−1^ body weight) or insulin (ITT; 0.75 U kg^−1^ body weight). Blood glucose levels at various times after the injections were determined by an electronic glucometer (Abbott Diabetes Care, Alameda, CA, USA). Lipids analysis and lipoprotein profile measurement were performed^60^. In brief, mice were fasted for 12–14 h before blood samples were collected by retro-orbital venous plexus puncture. Plasma was separated by centrifugation and stored at −80 °C. Total plasma cholesterol, TGs and FFAs were enzymatically measured with the Amplex red cholesterol assay kit (Molecular Probes), serum triglyceride determination kit and free fatty acid kit (Sigma), respectively, according to the manufacturer’s instructions. The lipid distribution in plasma lipoprotein fractions were assessed by fast-performance liquid chromatography gel filtration with two Superose 6 HR 10/30 columns (Pharmacia).

### Islet isolation and primary culture

Pancreatic islets were isolated from 3-month-old Ctrl and KO mice by collagenase P (Roche Applied Science) digestion^61^. Islets were picked by hand four times in succession under a dissecting microscope and cultured overnight in RPMI 1640 supplemented with 5 mmol l^−1^ glucose, 10% fetal bovine serum (FBS), 10 mmol l^−1^ HEPES, 2 mmol l^−1^ L-glutamine, 1 mmol l^−1^ sodium pyruvate, 50 μmol l^−1^ β-mercaptoethanol, 100 units penicillin per ml and 100 μg streptomycin per ml. For islet apoptosis assay, islets were isolated from the Ctrl mice and incubation for 16 h to recover from the harsh process of collagenase digestion. Islets (100 islets per well) were cultured with the serum from Ctrl, SENP1-aP2KO, NOD mice or human T1DM patients for 72 h. Islet viability was assessed by Live/Dead Viability/Cytotoxicity Kit (Molecular Probe).

### Cell lines and isolation of mature adipocytes

3T3-L1, a commonly used preadipocyte mouse cell line, was obtained from ATCC (CL-173) and cultured under DMEM media supplemented with 2 mM glutamine and 10% calf serum. The cell line validation by karyotyping and mycoplasma testing was performed before shipping from ATCC. Differentiation of 3T3-L1 to adipocyte was performed according to the protocol provided by the manufacturer. In brief, cells were cultured at sub-confluent (70–80%) 1:50 to 1:100, that is, seeding at 2–4 × 10,000 cells per cm^2^ in a 75-cm^2^ flask using 0.25% trypsin or trypsin/EDTA; 5% CO_2_; 37 °C. Two days after confluency differentiation was induced by adding 0.5 mM 3-isobutyl-1-methylxanthine, 0.25 μM dexamethoasone and 1 μg ml^−1^ insulin in DMEM with 10% FBS. After 2 days, the 3-isobutyl-1-methylxanthine and dexamethasone, but not insulin, were removed in culture for another 2 days. On day 4, differentiated cells were cultured in DMEM with 10% FBS and media were changed every other day for 2–4 weeks. The differentiated cells were incubated in serum-free DMEM for 2 h before experiments.

For primary adipocyte culture, adipose tissue was dissected from peri-pancreas or epididymal fat and incubated in Krebs-Ringer phosphate buffer (pH 7.4) containing 100 U ml^−1^ collagenase and 40 mg ml^−1^ BSA in a shaking bath at 37 °C for 1 h. The adipocytes were filtered through a polypropylene mesh (pore size 250 μm) and washed three times in Krebs-Ringer phosphate buffer with 0.1% BSA. Macrophage and T cells were labelled with phycoerythrin (PE)-CD11b and PE-CD3e antibodies and depleted with magnetically labelled PE antibody using an EasySep magnet (Stem Cell Technologies). The cells were incubated in DMEM/F-12 containing BSA at the concentrations of 300 mM for 24 h at 37 °C in 5% CO_2_ (ref. 62). For isolation pancreatic macrophage or T cells, PE-CD11b or PE-CD3e antibodies labelled cells were collected with magnetically labelled PE antibody using an EasySep magnet (Stem Cell Technologies).

### Clinical specimens

Ethical evaluation for the clinical specimens, and blood draws, from consenting human volunteers was reviewed and approved by the Yale human Investigation Committee (IHC#1005006865). Venous blood was drawn from volunteers (healthy and diabetic subjects) after obtaining consent at Yale University School of Medicine. Twenty-nine subjects with T1DM (American Diabetes Association definition) and 21 healthy control subjects were recruited for the studies based on established exclusion/inclusion criteria (glucose levels, body mass index, HbA1c and so on; Supplementary Table 1). Deidentified plasma was obtained from whole blood by centrifugation and immediately frozen at −80 °C.

### Insulin autoantibodies ELISA

Plasma IAA levels were analysed with mouse IAA ELISA kit (Biorbyt, CA, USA). Plasma samples (1:2 dilutions) were incubated in 96-well plates precoated with IAA antibody overnight. After washing, FITC-labelled IAA detection antibody was added for 1 h. FITC fluorescence intensity of bound antibody was detected with a MRX microplate reader (Dynex Technologies, VA, USA).

### Flow cytometer

Cells from pancreases were isolated from both SENP1-aP2KO and Ctrl mice. Immune cells were detected using LSRII flow cytometer (BD Biosciences, San Jose, CA, USA) and the FlowJo software (Tree Star Inc., Ashland, OR, USA)^63^. Surface staining was performed using T-cell markers (CD3e, CD4 and CD8), Mac/DC markers (MHCII, CD11c, CD11b and CD68) and the B-cell marker CD19 (BD). Intracellular staining was performed for adipocyte (Nile, IL-6, TNF-α, IFNγ and IL-1β), T-cell subset Th1 (CD3e, CD4 and IFNγ), Th17 (CD3e, CD4 and IL-17) and regulatory T cell (CD4, CD25 and Foxp3). Isotype controls were used in each experiment.

### Monitoring of antigen-specific CD8 T cells

CD8^+^ T cells were isolated from spleen and pancreatic lymph nodes of Ctrl and KO mice and staining with H-2Kd NRP-V7 Tetramer-KYNKANVFL-Allophycocyani (APC) (MHC Tetramer Core Facility, Atlanta, GA, USA). In addition, an APC-labelled negative tetramer H-2Kd TUM Tetramer-KYQAVTTTL-APC was used as a control to assess the level of background fluorescence and nonspecific binding.

### β-Cell apoptosis analysis

Paraffin-embedded pancreases were sectioned at 7-μm intervals and subjected to TUNEL staining using an *in situ* cell death detection kit (Roche Diagostics, Indianapolis, IN, USA). For islet cells, this reaction was blocked with a blocking buffer, and the slides were immunostained with rabbit anti-insulin (β cell) and goat anti-glucagon (α-cell; Cell Signaling Tech, Danvers, MA, USA) for 2 h and then with donkey-derived secondary antibodies conjugated to PE (Invitrogen, Carlsbad, CA, USA). All slides were mounted with 4′,6-diamidino-2-phenylindole-containing medium. Fluorescent slides were viewed using a Leica DM6000 microscope, and images were acquired using software. Apoptosis was analysed by counting the TUNEL-positive β cells in each islet. Alternatively, islets were isolated from pancreases, β cells in islets were gated as insulin positive cells with a flow cytometer and apoptotic cells were identified as TUNEL-positive cells.

### Chromatin immunoprecipitation assay

The ChIP assay was performed using ChIP kit (Millipore; #17–371) with p65/RelA antibody (SC-109; 1:40 dilution, 5 μg ml^−1^) and analysed by real-time PCR. All the primers are listed in Supplementary Table 2. Normal IgG (SC-2027; 1:40 dilution, 5 μg ml^−1^) was used as an isotype control. Anti-RNA polymerase II (Millipore, #05–623B; 1:500, 2 μg ml^−1^) was used as a positive control.

### Quantitative real-time PCR

Total RNA was isolated from mouse tissues with Trizol Reagent (Invitrogen). Reverse transcription was performed by a standard procedure (Super Script First-Strand Synthesis System; Invitrogen) using 1 μg of total RNA. Quantitative real-time PCR was performed using iQ SYBR Green Supermix on an iCycler Real-Time Detection system (Bio-Rad, Hercules, CA, USA). Specific primers for mouse cytokines, chemokines, SENP1 and GAPDH genes were used. The relative amount of mRNA was normalized with a housekeeping GAPDH as indicated and was quantified^60^. Real-time PCR with reverse transcription reactions were also performed using TaqMan 2 × PCR Master Mix, TaqMan PCR reagents and commercially available TaqMan gene expression probes for chemokines or hypoxanthine-guanine phosphoribosyltransferase. Standard curves with known concentration of template copy numbers were used to determine the expression of amplified targets. The expression level of each target gene was normalized to hypoxanthine-guanine phosphoribosyltransferase and the results were given as relative copy numbers^60^.

### Haematoxylin and eosin staining and immunohistochemistry

Paraffin-embedded tissues were sectioned at 7-μm intervals, and the sections were subjected to standard haematoxylin and eosin staining or immunostained using primary antibodies, with anti-CD8, anti-CD4, anti-CD31, anti-CCL5, anti-insulin, anti-FABP4, anti-p-p65/RelA and anti-F4/80, with isotype-matched primary antibodies serving as controls. Antibody binding was visualized with fluorescence-labelled secondary antibodies and examined by microscopy. The sections that were paraffin-embedded were dewaxed before being treated with anti-mouse antibodies.

### RNA interference for SENP1

In view of the established characteristics of siRNA-targeting constructs, we designed three pairs of siRNA oligonucleotides for SENP1: siRNA 21696: 5′-GGAAAUGGAGAAAGAAAUA dTdT-3′; siRNA21512: 5′-GGACCAGCUUUCGCUUUCU dTdT-3′; siRNA21605: 5′-GGACAUUUGGACCGAUCUU dTdT-3′. Three siRNAs were obtained similar knockdown efficiency. The corresponding scramble siRNA oligonucleotide for siRNA21512: 5′-GGA CCA GCA UAC GCU UUCU dTdT-3′, with two nucleotide mutations (underlined), was synthesized from Ambion (Austin, TX, USA). siRNAs (20 μM) were transfected into cells by Oligofectamine (Life Technologies, Inc.; Invitrogen), following protocols provided by the manufacturer (Invitrogen). At 72 h post transfection, cells were collected for analysis.

### SENP1 plasmid construct and gene overexpression

Expression plasmids for GFP-tagged SENP1 were generated by inserting mouse SENP1 into pEGFP-C2. Adipocytes were transfected by Lipofectamine 2000. SENP1 vectors (1 μg) were transfected into cells by Oligofectamine, following protocols provided by the manufacturer. At 48 h post transfection, cells were collected for protein analysis.

### Immunoprecipitation and immunoblotting

Adipocytes were washed twice with cold PBS and lysed in 1.5 ml of cold lysis buffer (50 mM Tris-HCl, pH 7.6, 150 mM NaCl, 0.1% Triton X-100, 1 mM sodium orthovanadate, 1 mM sodium fluoride, 1 mM sodium pyrophosphate, 10 mg ml^−1^ aprotinin, 10 mg ml^−1^ leupeptin, 2 mM phenylmethylsulphonyl fluoride and 1 mM EDTA) on ice. For detection of SUMOylated proteins, 1 mM *N*-ethylmaleimide (deSUMOylase inhibitor) was added to cell lysates. For western blotting, proteins were detected by SENP1, p65/RelA, IκBα and ERK1/2, pIκBα, p-p65/RelA, pIKKα/β, IKKβ and pERK1/2 antibodies, and secondary antibodies, with the use of a chemiluminescent detection system (Amersham Life Science, Arlington Heights, IL, USA). To ensure equal loading, membranes were stripped and reprobed for β-actin using anti-actin antibodies. For protein SUMOylation assay, adipocytes were washed twice with cold PBS and lysed in denaturing lysis buffer (1% SDS, 5 mM EDTA, 10 mM dithiothreitol, protease inhibitors 15 U ml^−1^ and DNase1). Cell lysates were heated at 95 °C for 10 min, followed by dilution with 0.9 ml nondenaturing lysis buffer. Supernatants of cell lysates were precleared by incubating with protein A/G PLUS-Agarose for 2 h. The lysates were then incubated with anti-NEMO or anti-p65/RelA, with 50 μl of protein A/G PLUS-Agarose on a rotator at 4 °C overnight. Immune complexes were collected after each immunoprecipitation by centrifugation at 13,000 *g* for 10 min, followed by washes with lysis buffer. The immune complexes were subjected to SDS–polyacrylamide gel electrophoresis, followed by immunoblotting with the SUMO1, NEMO or p65/RelA-specific antibodies. All antibodies used are listed in Supplementary Table 3. Uncropped scans of the original western blots are shown in Supplementary Fig. 8.

### Site-directed mutagenesis of SUMOylation sites K277 and K309 of NEMO

The SUMOylation sites of NEMO were mapped to the positions K277 and K309. The pcDNA flag-NEMO K277 and/or K309 mutation expression vectors were generated using a pcDNA flag-NEMO-WT template and the QuickChange XL-II Site-directed Mutagenesis Kit (Stratagene, La Jolla, CA, USA).

### Study design and statistical analysis

Group sizes were determined by an *a priori* power analysis for a two-tailed, two-sample *t*-test with an *α* of 0.05 and power of 0.8, to detect a 10% difference in diabetic parameters (for example, glucose level, insulin level, lipid profile, body weight and cytokines) at the end point. Animals were grouped with no blinding but randomized during the experiments. No samples or animals were excluded from analysis. All quantifications (for example, glucose level, insulin level, lipid profile, body weight, cytokines, apoptosis, immune cell populations, NF-κB activation, infiltration and islet disruption) were performed in a blind manner. All figures are representative of at least three experiments unless otherwise noted. All graphs report mean±s.e.m. values of biological replicates. Comparisons between two groups were performed by paired *t*-test, between more than two groups by one-way analysis of varinace followed by Bonferroni’s *post hoc* test or by two-way analysis of varinace using Prism 4.0 software (GraphPad). *P* values were two-tailed and values <0.05 were considered to indicate statistical significance. *P*<0.05, *P*<0.01 and *P*<0.001 are designated in all figures unless specified with *, ** and ***, respectively.

## Additional information

**How to cite this article:** Shao, L. *et al.* SENP1-mediated NEMO deSUMOylation in adipocytes limits inflammatory responses and type-I diabetes progression. *Nat. Commun.* 6:8917 doi: 10.1038/ncomms9917 (2015).

## Supplementary Material

Supplementary Information Supplementary Figures 1-8, Supplementary Tables 1-3.
