# Author Correction: SENP1-mediated NEMO deSUMOylation in adipocytes limits inflammatory responses and type-1 diabetes progression

**DOI:** 10.1038/s41467-025-60998-6

**Published:** 2025-06-23

**Authors:** Lan Shao, Huanjiao Jenny Zhou, Haifeng Zhang, Lingfeng Qin, John Hwa, Zhong Yun, Weidong Ji, Wang Min

**Affiliations:** 1 The First Affiliated Hospital, Center for Translational Medicine, Sun Yat-sen University, Guangzhou, China; 2 Department of Pathology, Interdepartmental Program in Vascular Biology and Therapeutics, Yale University School of Medicine, 10 Amistad St, New Haven, Connecticut 06520 USA; 3 Department of Internal Medicine and Section of Cardiology, Yale University School of Medicine, New Haven, Connecticut 06510 USA; 4 Department of Therapeutic Radiology, Yale University School of Medicine, New Haven, Connecticut 06510 USA

Correction to: *Nature Communications* 10.1038/ncomms9917, published online 24 November 2015

In the version of the article initially published, in Supplementary Fig. 4e, the “aP2KO, Lung” image was a duplicate of the “Ctrl, Lung” image. The “aP2KO, Lung” image has been corrected and can be found in the Supplementary information accompanying this amendment.

## Supplementary information

Corrected Supplementary information

