# Supplementary material for: SENP1-mediated NEMO deSUMOylation in adipocytes limits inflammatory responses and type-1 diabetes progression
Source: Nat Commun. 2015 Nov 24;6:8917. doi: 10.1038/ncomms9917 (PMC4662081; doi:10.1038/ncomms9917)
Supplement: Supplementary Information — Supplementary Figures 1-8, Supplementary Tables 1-3. [file ncomms9917-s1.pdf]

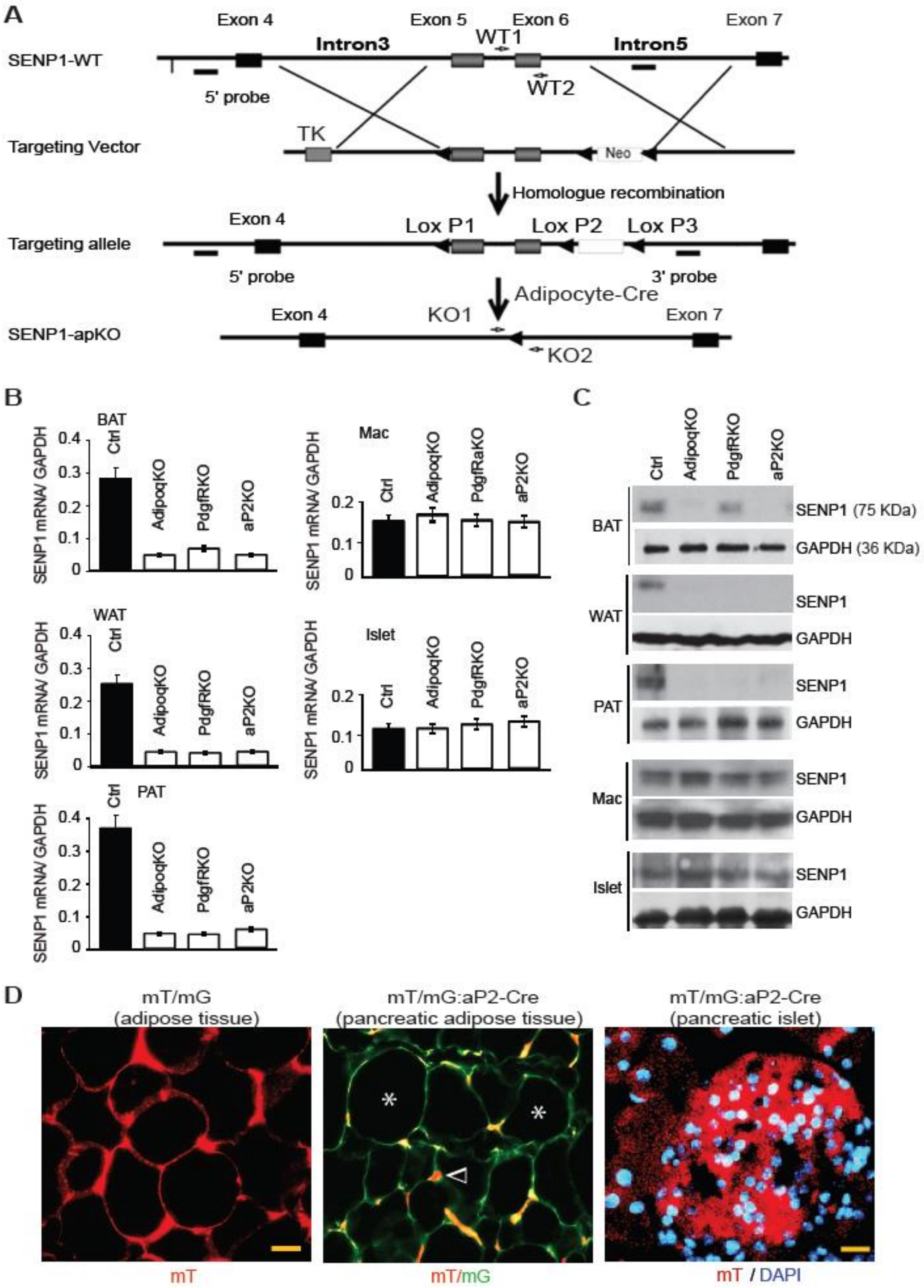

Supplementary Fig.1. Specific deletion of SENP1 in adipocytes of SENP1-deficient mice.

(A) SENP1$^{lox/lox}$ mice were generated based on homologous recombination. SENP1$^{lox/lox}$ mice were mated with adipocyte specific Cre to obtain adipocyte-specific SENP1-deficient (SENP1-apKO) mice.

(B-C) SENP1 is deleted in adipocytes. Adipocytes were isolated from SENP1$^{lox/lox}$ Ctrl, SENP1-AdipoqKO, SENP1-PdgfRKO and SENP1-aP2KO mice (n=3 male in each group). SENP1 protein expression in BAT, WAT, PAT, Macrophage (Mac) and Islet were detected by qRT-PCR (B) and Western blot (C), respectively. SENP1 is intact in the macrophage and islets of SENP1-deficient mice. qRT-PCT data in are shown as means ± SEM, n=3, male. A representative immunoblot from one of 3 mice is shown.

(D) aP2-Cre mice were crossed with a genetic Cre reporter ROSA-26Sor$^{tm4(ACTB-tdTomato,\ EGFP)Luo}$/J (mT/mG mice). Cells expressing aP2-Cre deleted the mT (red) cassette, allowing expression of the membrane-targeted EGFP (mG) cassette located downstream. EGFP was not detected in adipose tissues of mT/mG mice (left panel). In mT/mG:aP2-Cre mice, EGFP expression was detected in the adipose tissue surrounding pancreas (indicated by *) but not in capillaries (indicated by an arrowhead) (middle panel) or the pancreatic islet (right panel). Scale bar: 20 μm.

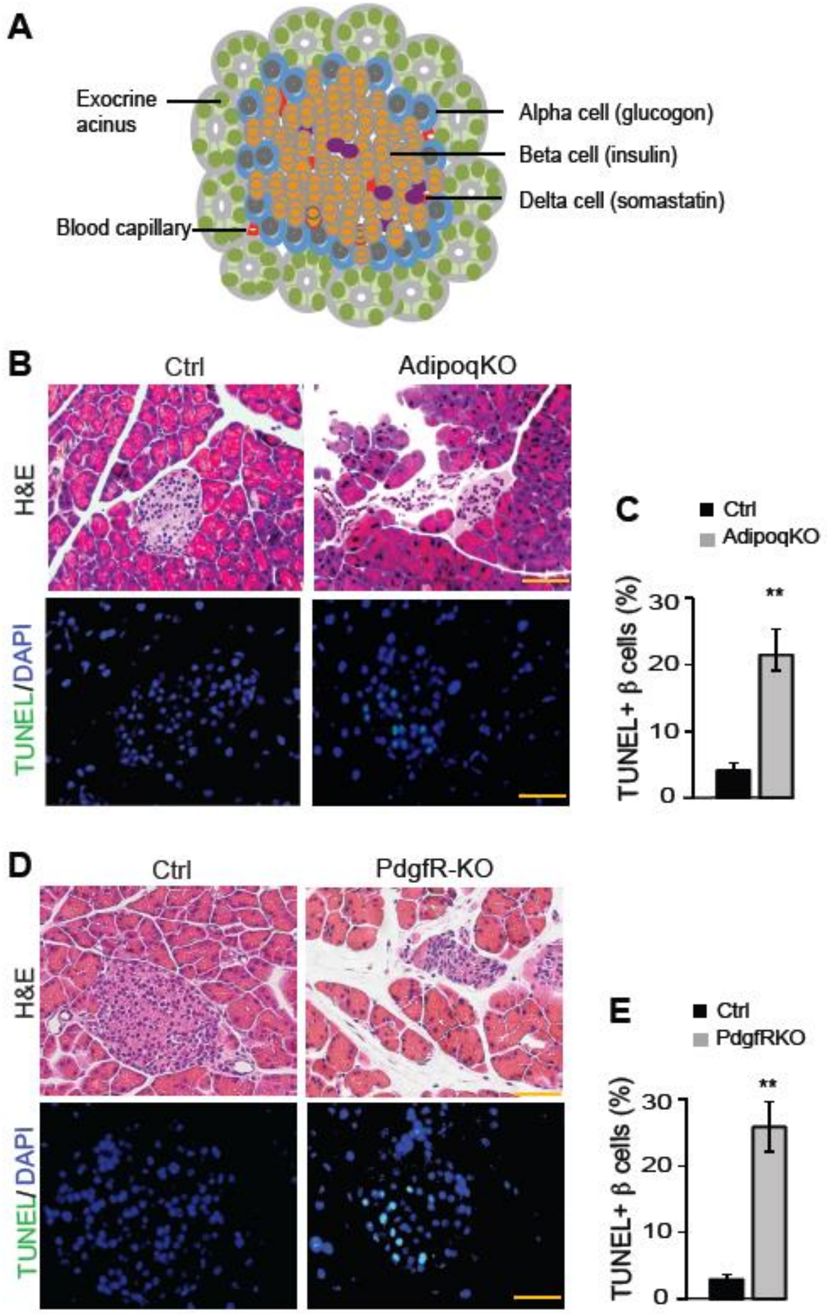

Supplementary Fig.2. Islet structure, β cell death and T cell infiltration in SENP1-deficient mice. (A) A schematic diagram for pancreatic islet. The major cell types and blood vessels are indicated. The β cells, which are the most common and account for 60-75% of the cells in the islets, are generally located in the center of each islet. Insulin from the β cell and glucagon from the α cell are secreted by exocytosis. (B-C) H&E staining for overall morphology of the pancreases and TUNEL in situ cell death staining for paraffin-embedded pancreases from 6 pairs age-matched Ctrl and SENP1-AdipoqKO male mice at age of 12 weeks. Scale bar: 20 μm. (D-E) H&E staining for overall morphology of the pancreases and TUNEL in situ cell death staining for paraffin-embedded pancreases from 6 pairs age-matched Ctrl and SENP1-PdgfRKO male mice at age of 12 weeks. Scale bar: 20 μm. Data in C and E are shown as means ± SEM, n=6. **, *P*<0.01.

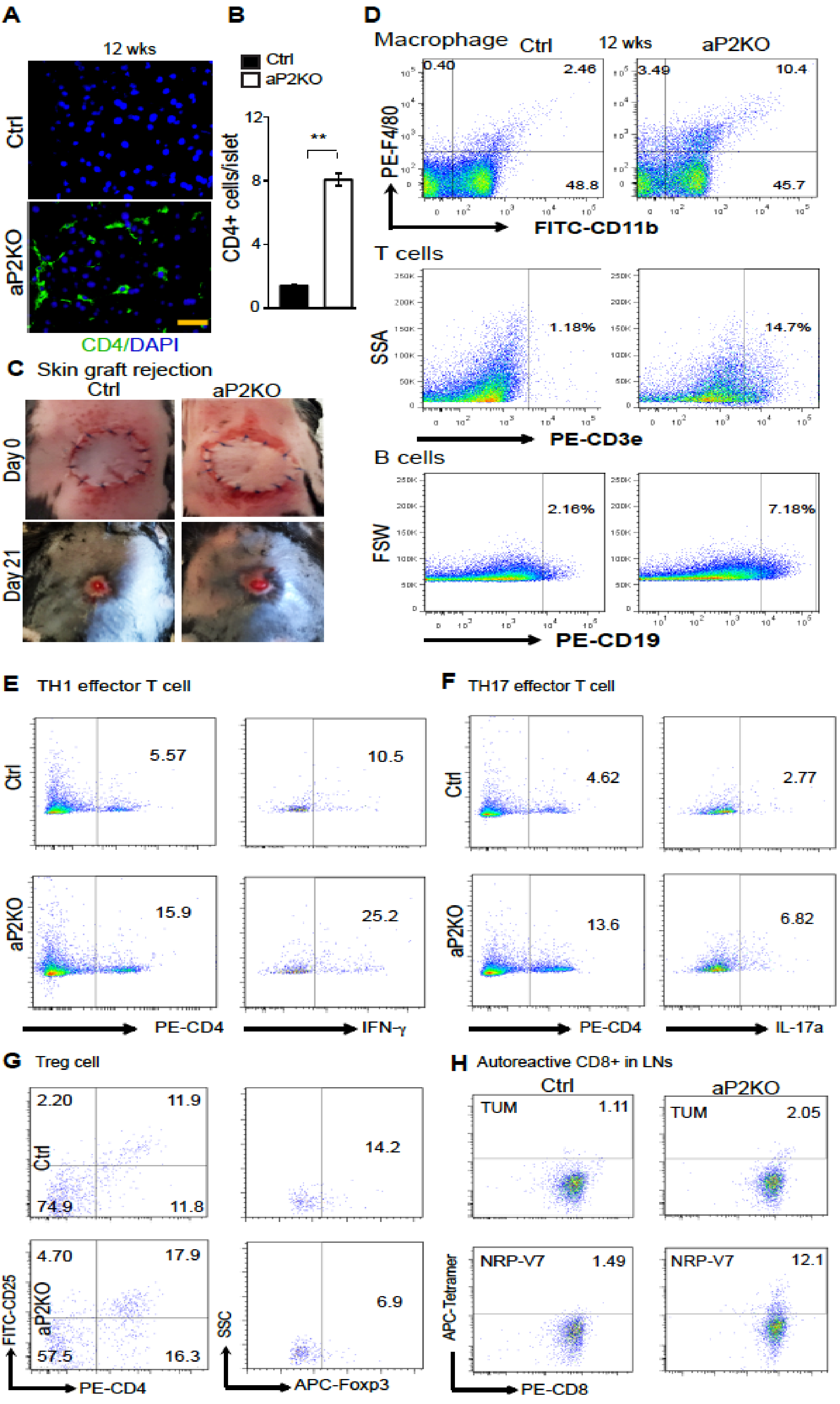

A
12 wks
Ctrl
aP2KO
CD4/DAPI
B
Ctrl
aP2KO
CD4+ cells/islet
**
C Skin graft rejection
Ctrl
aP2KO
Day 0
Day 21
D
Macrophage
Ctrl
12 wks
aP2KO
PE-F4/80
FITC-CD11b
0.40
2.46
48.8
3.49
10.4
45.7
T cells
SSA
PE-CD3e
1.18%
14.7%
B cells
FSW
PE-CD19
2.16%
7.18%
E TH1 effector T cell
5.57
10.5
15.9
25.2
PE-CD4
IFN-γ
F TH17 effector T cell
4.62
2.77
13.6
6.82
PE-CD4
IL-17a
G Treg cell
2.20
11.9
74.9
11.8
14.2
4.70
17.9
57.5
16.3
6.9
FITC-CD25
PE-CD4
SSC
APC-Foxp3
H Autoreactive CD8+ in LNs
TUM
1.11
TUM
2.05
NRP-V7
1.49
NRP-V7
12.1
APC-Tetramer
PE-CD8

Supplementary Fig.3. Characterization of immune responses in SENP1-deficient mice.

(A-B) CD4 T cell infiltrations in pancreases of SENP1-aP2KO mice at age of 12 weeks. Representative images of frozen pancreases sections from Ctrl and SENP1-aP2KO mice were stained with anti-CD4 antibody (green). Scale bar: 20 μm. Numbers of $CD4^+$ T cells invading into the islet of Ctrl and SENP1-aP2KO mice were quantified at B. Data are presented as means ± SEM from 6 paired samples of Ctrl and SENP1-aP2KO mice (n=6, male). **, P<0.01. (C) Skin from BALB/C background mice was graft to SENP1lox/lox and SENP1-aP2KO mice (B6 background). Severe skin-rejection immune response appeared in both Ctrl and aP2KO mice at day 21 after transplantation, indicating there is no immuno-deficiency in SENP1-aP2KO mice. Representative image from one pair of Ctrl and SENP1-aP2KO mice are shown. Similar results were obtained from additional five pairs of mice. (D) Total macrophages ($CD11b^+F4/80^+$), T cells ($CD3^+$) and B cells ($CD19^+$) in pancreatic adipose tissue of Ctrl and SENP1-aP2KO mice (n=6 each) were detected by FACS at the age of 12 weeks. (E-G) Different type of effector T cells distribution in pancreases. Representative FACS images of Th1 (IFN-γ^+ CD3+CD4+) effector T cells (E), Th17 (IL-$17A^+$CD3+CD4+) effector T cells (F) and regulatory T cells (CD4+CD25+Foxp3) (G) in pancreases of Ctrl and SENP-aP2KO mice. (H) CD8+ T cells isolated from pancreatic lymph node of 15-week-old Ctrl and KO mice were stained with TUM (Ctrl) and islet autoantigen (NRP-V7)-specific tetramers and then detected by FACS. Data are presented as means from 6 paired samples of Ctrl and SENP1-aP2KO male mice.

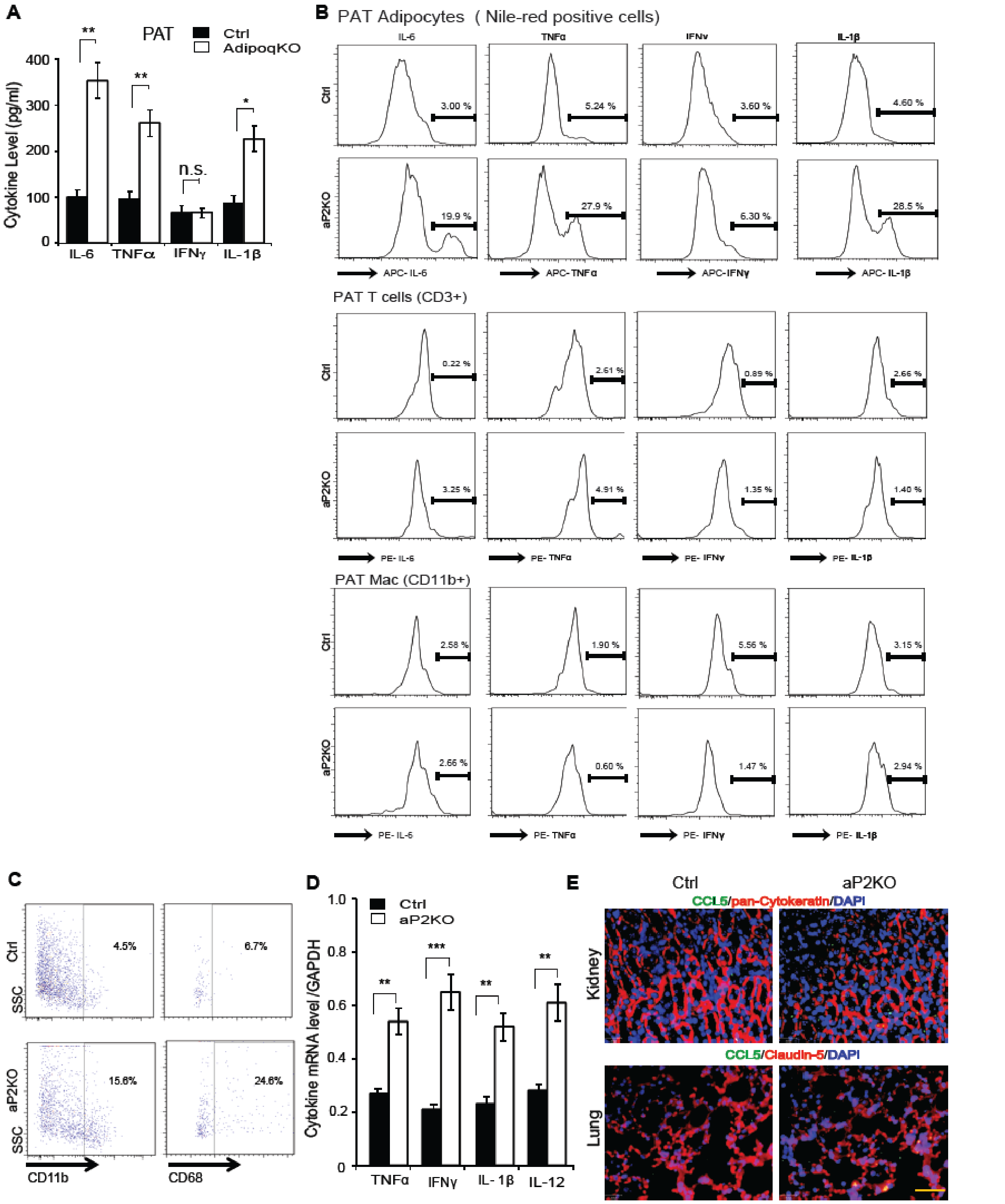
A
PAT
Ctrl
AdipoqKO
Cytokine Level (pg/ml)
IL-6
TNFα
IFNγ
IL-1β
n.s.
B
PAT Adipocytes (Nile-red positive cells)
APC- IL-6
APC- TNFα
APC- IFNγ
APC- IL-1β
PAT T cells (CD3+)
PE- IL-6
PE- TNFα
PE- IFNγ
PE- IL-1β
PAT Mac (CD11b+)
Ctrl
aP2KO
C
SSC
CD11b
CD68
D
Cytokine mRNA level /GAPDH
IL-12
E
CCL5/pan-Cytokeratin/DAPI
CCL5/Claudin-5/DAPI
Kidney
Lung

Supplementary Fig.4. Effects of SENP1 deletion on cytokine expression in PATs and CCL5 induction in pancreatic islets.

(A) Protein levels of IL-6, TNF-α, IFN-γ and IL-1β levels present in culture supernatant of isolated pancreatic adipocytes in Ctrl and SENP1-AdipoqKO with ELISA after 24 h culture. (B) Intracellular cytokine expression in PAT adipocytes. Adipocytes were labeled with Nile red staining. IL-6, TNFα, IFNγ, IL-1β expression levels in Nile red positive adipocytes, $CD3^+$ T cells and $CD11b^+$ macrophages were detected with intracellular staining by FACS with % of positive cells indicated. (C). Macrophage phenotype at later phases. Total macrophages ($CD11b^+$) and activated macrophage ($CD11b^+CD68^+$) in PAT of Ctrl (n=6) and SENP1-aP2KO (n=6) male mice were detected by FACS at the age of 14 weeks. %$CD11b^+$/total PAT cells and % $CD68^+/CD11b^+$ cells are shown. (D) Gene expression of TNF-α IFN-γ, IL-1β and IL-12 levels in the pancreases of Ctrl and SENP1-aP2KO were detected by qRT-PCR at the age of 14 weeks. (E) CCL5 expression. Sections of kidney and lung from Ctrl and SENP1-aP2KO mice were co-immunostaining with CCL5 (green) and pan-cytokeratin (kidney) or CCL5 and endothelial marker claudin-5 (lung). Representative images of the kidney and lung from Ctrl and SENP1-aP2KO mice at age of 7 weeks are shown, n=6. Scale bar: 20 μm. All data are means± SEM from n=6 male mice per group. *, $P<0.05$; **, $P<0.01$; ***, $P<0.001$; n.s: no significance.

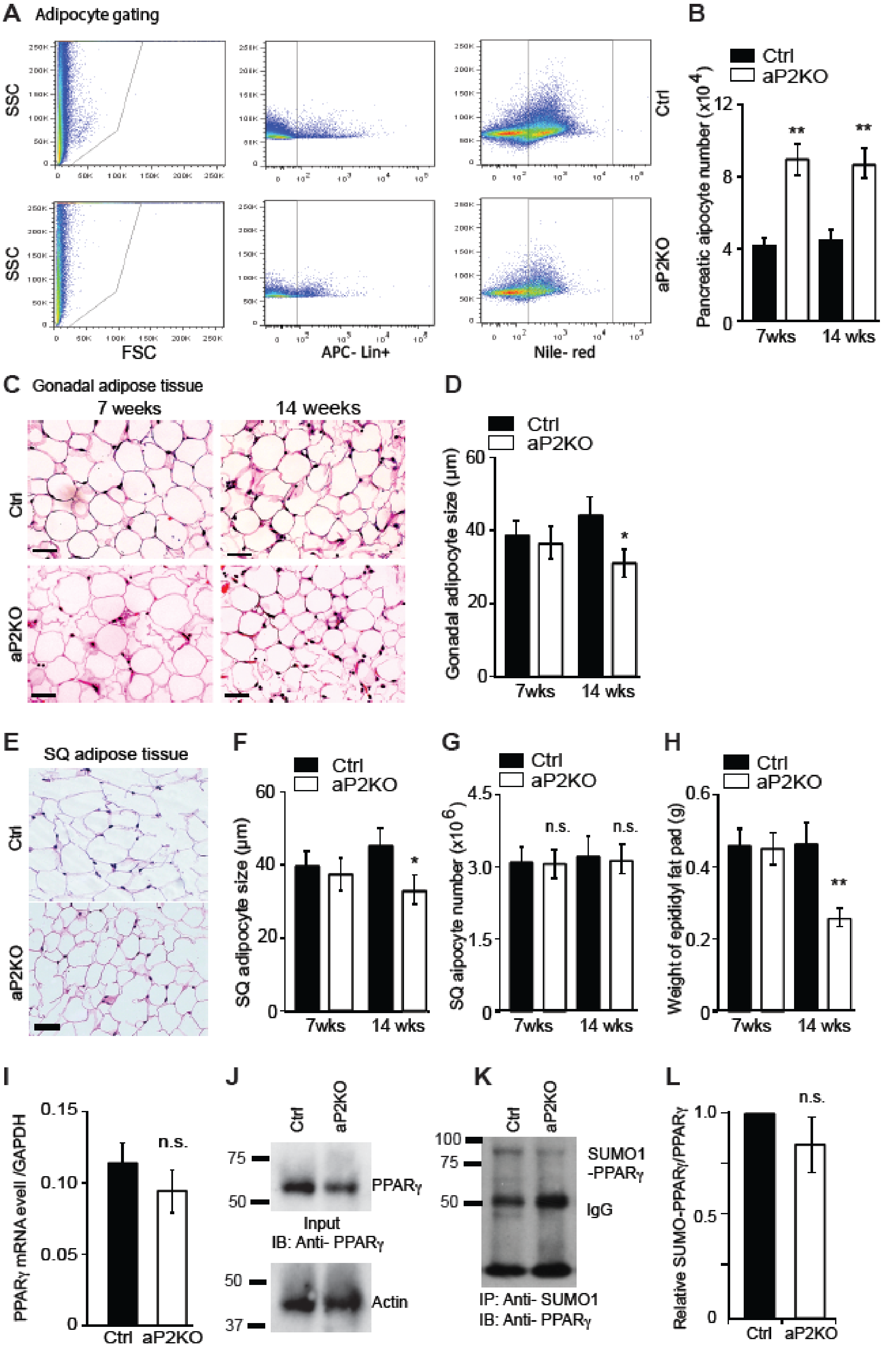
A Adipocyte gating
SSC
FSC
APC- Lin+
Nile- red
Ctrl
aP2KO
B
Ctrl
aP2KO
Pancreatic aipocyte number (x10^4)
7wks
14 wks
C Gonadal adipose tissue
7 weeks
14 weeks
D
Gonadal adipocyte size (μm)
E
SQ adipose tissue
F
SQ adipocyte size (μm)
G
SQ aipocyte number (x10^6)
n.s.
H
Weight of epididyl fat pad (g)
I
PPARγ mRNA evell /GAPDH
J
PPARγ
Input
IB: Anti- PPARγ
Actin
K
SUMO1
-PPARγ
IgG
IP: Anti- SUMO1
IB: Anti- PPARγ
L
Relative SUMO-PPARγ/PPARγ

Supplementary Fig.5. Characterization of adipose tissues in SENP1-deficient mice.

(A) Representative FACS images for measurements of adipocyte cell numbers by FACS with Nile-red staining. Adipocytes are gated by Lin^- and $nile\text{-}red^+$ staining. Note: SENP1-aP2KO adipocytes have lower FSC value than Ctrl pancreatic adipocytes, suggesting SENP1-aP2KO adipocytes are smaller in sizes. SENP1-aP2KO adipocytes exhibit weaker Nile-red staining than Ctrl, likely due to less mature with reduced lipid drops of SENP1-aP2KO adipocytes. (B) Pancreas adipose tissues. PATs were collected from 7 and 14 weeks old of Ctrl and SENP1-aP2KO male mice. Adipocytes numbers were measured by FACS with Nile-red staining. (C-D) Gonadal adipose tissue (GAT). GAT morphology at week 7 and 14 were visualized by H&E staining (C) and adipocyte size with quantifications were analyzed at (D). Scale bar: 20 μm. (E-F) Subcutaneous adipose tissue (SAT). SAT were collected from 14 weeks old of Ctrl and SENP1-aP2KO male mice. The morphology of adipose tissues were visualized by H&E staining (E) and adipose size were measured at (F). (G) The numbers of subcutaneous adipocytes from Ctrl and SENP1-aP2KO mice were measured by FACS with Nile-red staining. (H) Epididymal fat pad from Ctrl and SENP1-aP2KO mice were weighted. (I-L) PPARγ expression. (I) Transcription levels of PPARγ from PAT of Ctrl and SENP1-aP2KO mice were measured by qRT-PCR with quantification. GAPDH was used for normalization. (J) PPARγ protein levels were detected with western blotting. (K) Proteins extracted from the adipocytes of Ctrl and SENP1-aP2KO mice at age of 5 weeks were subjected to immunoprecipitation with SUMO1 antibody followed by Western blotting with anti-PPARγ. Representative blots from one pair of Ctrl and SENP1-aP2KO mice are shown. Similar results were obtained from additional five pairs of mice. (L) The ratio of SUMO-PPARγ/PPARγ was calculated according J and K. All data are means ± SEM from n=6 mice per group. *, $P<0.05$; n.s: no significance.

.

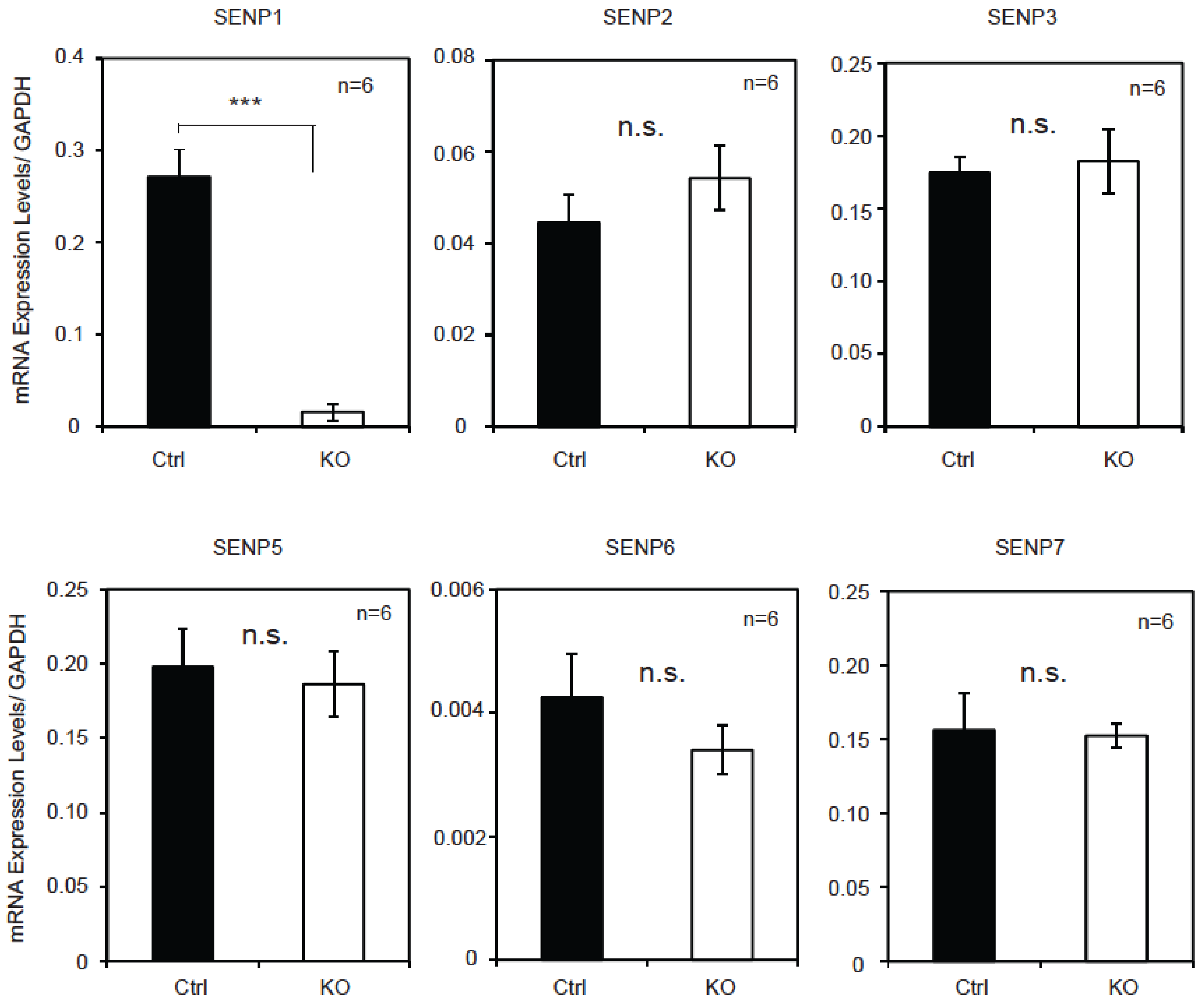

Supplementary Fig.6. A. Expression of SENPs in mouse adipose tissues.

mRNAs were extracted from adipose tissues of Ctrl and SENP1-aP2KO male mice at age of 5 weeks. Transcript levels of SENP1, SENP2, SENP3, SENP5, SENP6 and SENP7 were quantified by qRT-PCR with specific primers. GAPDH was used for normalization. SENP1 was not detected in SENP1-KO adipose tissue. SENP1 deletion had no effects on the gene expression of other members of SENPs. All data are means ± SEM from n=6 male mice per group. ***, *P*<0.001; n.s: non significance.

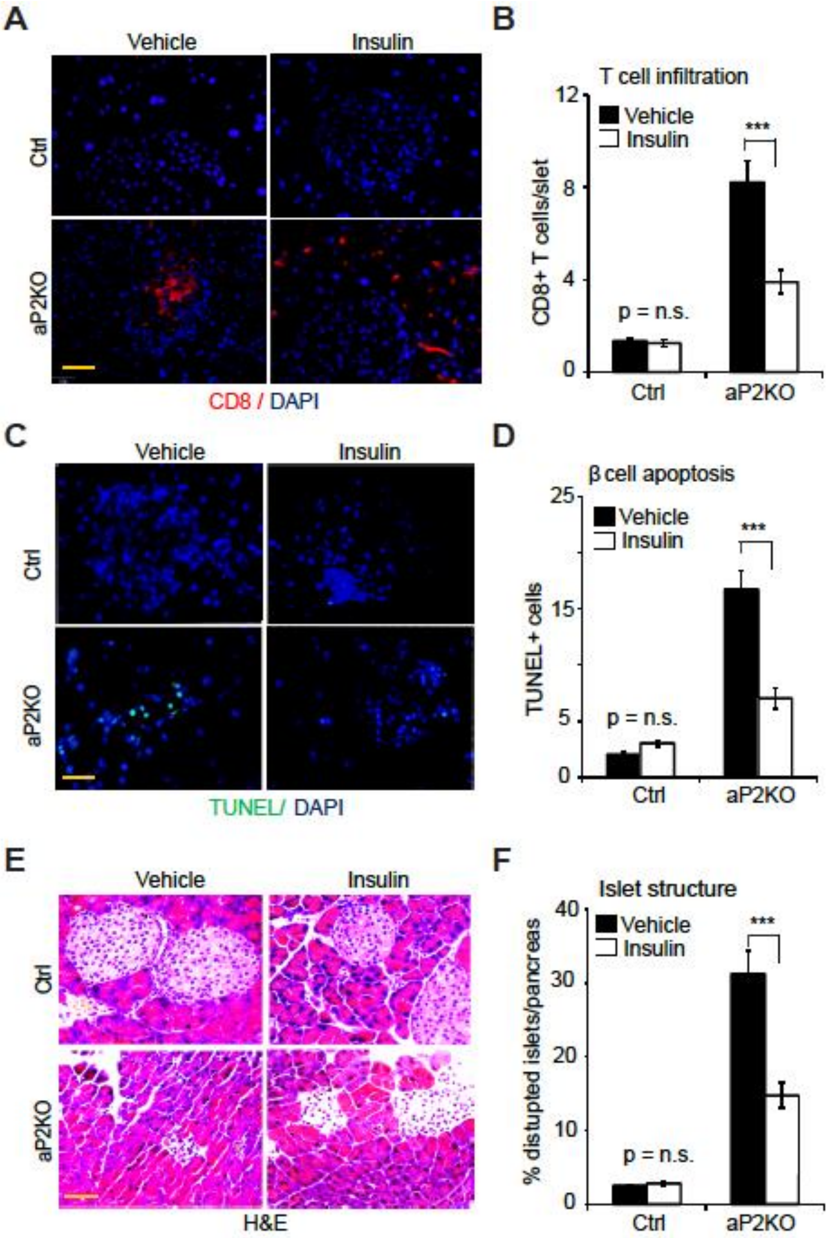

Supplementary Fig.7. **Insulin responses in SENP1-deficient mice**.

Ctrl and SENP1-aP2KO mice were treated with insulin (0.5 U/mouse per day) at age of week 5 for 9 weeks (n=6, each group). (A-B) T cell infiltration. Representative images were anti-CD8 staining (red) of Ctrl and SENP1-aP2KO mice at age of 14 weeks and numbers of $CD8^+$ T cells invading into the islet of Ctrl and SENP1-aP2KO mice were quantified. (C-D) TUNEL staining together with an anti-insulin antibody was performed. $TUNEL^+$ β-cells were quantified. (E-F). HE staining for whole morphology of pancreases and % of disrupted islet in the pancreases were quantified. Scale bars: 20 μm. Data are means ± SEM of 5 sections from each islet, n=6 male mice per group at each age. ***, *P*<0.001, n.s: non significance.

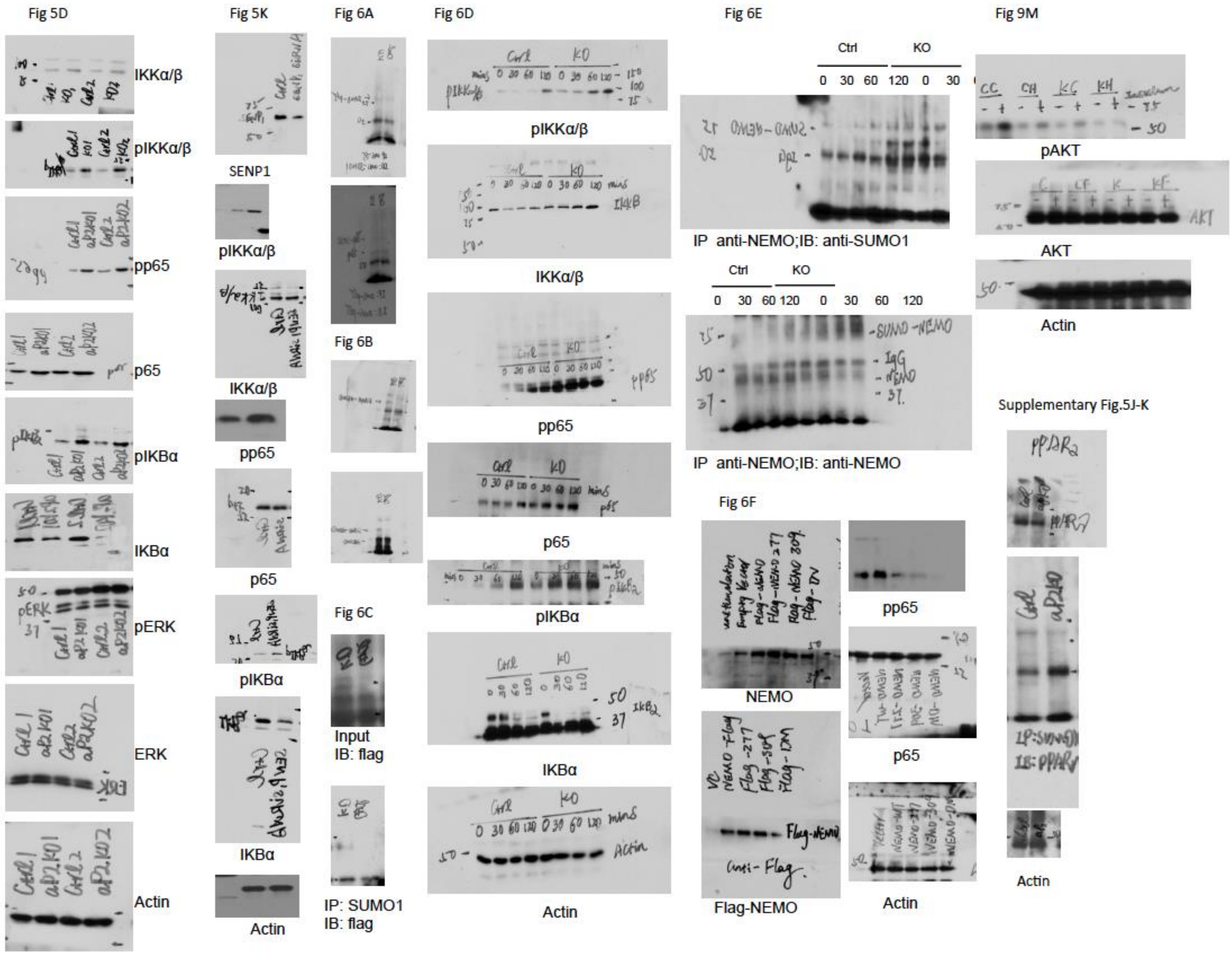

Supplementary Fig.8. **Uncropped scans of the original Western blots.**

Supplementary Table 1. Characteristics of recruited patients and controls

	Control	TIDM	P Value
**Characteristics**			
Number of subjects	21	29	0.51
Female/Male[a]	7/14	11/17	0.22
Age (mean ± SD years)[a]	56.1 ± 13.2	53.36 ± 14.5	0.31
**Ethnicity [a]**			
African American	13.1%	10.2%	
Caucasian	76.9%	82.8%	
Asian	10.1%	7.0%	
**Disease duration(mean ± SD years)**	0	11.3 ± 6.4	
**Glycaemia**			
Fasting glucose (mg/ dL)	95.2 ± 5.8	147.2± 52.1	
Glycated hemoglobin(H1bA1c%)	5.4 ± 0.3	7.7 ± 1.3	
**Diabetic risk factors**			
Family history of diabetes, n%	3%	51.7%	
Body mass index(kg/m^2)	21.8 ± 2.1	26.6 ± 4.8	
Hypertension	0	10.3	
HDL cholesterol (mg/dL)	63.5 ± 12.7	55.8 ±15.7	
LDL cholesterol (mg/dL)	109.6 ± 27.4	116.4 ±34.7	
Triglycerides (mg/dL)	88.5 ± 22.3	98.6 ± 39.2	
**Medications**			
Insulin	0	72.1%	
Metformin	0	20.7%	
Aspirin	0	51.2%	
Statin	0	41.3%	

[a] No significant difference

Supplementary Table 1. Characteristics of recruited patients and controls. Venous blood was drawn from consenting volunteers (healthy and diabetic subjects) at Yale University School of Medicine (Human Investigation Committee No. 1005006865). 29 subjects with type 1 DM (American Diabetes Association definition) and 21 healthy control subjects were recruited for the studies based on established exclusion/inclusion criteria (glucose levels, BMI, HbA1c, and etc.). De-identified plasma was obtained from whole blood by centrifugation and immediately frozen at -80°C. Age-matched Ctrl (n=21) and T1DM patients (n=29) were used in this study.

Supplementary Table 2. Primers for ChIP assays

IL-6 promoter:

Sense: 5′ -AGCTACAGACATCCCCAGTCTC-3′

Anti-sense: 5′ -TGTGTGTCGTCTGTCATGCG-3′

TNF–α promoter:

Sense: 5′ -CCCCAGATTGCCACAGAATC-3′

Anti-sense: 5′ -CCAGTGAGTGAAAGGGACAG-3′

IL-1β promoter:

Sense: 5’ -CCC CTA AGA ATT CCC ATC AAG C-3’

Anti-sense: 5’ -GAG CTG TGA AAT TTT CCCTTG G-3’

Supplementary Table 3: List of Antibodies used in this study

Antibody name	Company	Cat#	Dilution
**Western blotting**			
SENP1 (y-20), goat	Santa Cruz	#46634	1:500
NF-κB p65, rabbit	Santa Cruz	#109	1:1000
pp65, rabbit	Cell Signaling Technology	#3033P	1:500
IKKα, rabbit	Cell Signaling Technology	#2682P	1:500
IKKβ (2C8), rabbit	Cell Signaling Technology	#2370	1:500
pIKKα/β, rabbit	Cell Signaling Technology	#2697P	1:500
IKBα(C-21), rabbit	Santa Cruz	#371	1:500
pIKBα(Ser 32), rabbit	Cell Signaling Technology	#2859	1:500
pERK, mouse	Cell Signaling Technology	#9106S	1:1000
ERK(K-23), rabbit	Santa Cruz	#94	1:1000
SUMO1 (n-19), goat	Santa Cruz	#6376	1:500
SUMO1(C9H1), rabbit	Cell Signaling Technology	#49408	1:500
NEMO (FL-419), rabbit	Santa Cruz	#8330	1:1000
NEMO (M-19), goat	Santa Cruz	#8256	1:1000
Akt 1/2/3 (H-136), rabbit	Santa Cruz	#8312	1:1000
pAkt (Ser 473)	Cell Signaling Technology	#9271	1:1000
PPARγ (H-100), rabbit	Santa Cruz	#7196	1:1000
Flag M2-peroxidase, mouse	Sigma	#A8592	1:1000
**Immunohistology**			
CD8, rabbit	Abcam	ab4055	1:300
CD4 (GK1.5), rat	Abcam	ab25475	1:300
CD31, rabbit	Abcam	ab28364	1:300
FABP4, rabbit	Cell Signaling	#3544	1:300

	Technology		
Insulin, rabbit	Cell Signaling Technology	#4590	1:300
Glucagon, rabbit	Cell Signaling Technology	#8233P	1:300
F4/80, rat	Abcam	ab6640	1:300
CCL5 (C-19), goat	Santa Cruz	#1410	1:100
**ELISA**			
Biotin-IL-6	BD Biosciences	#554402	1:500
IL-6	BD Biosciences	#554400	1:500
Biotin-TNFα	eBioscience	#13-7326-85	1:500
TNFα	eBioscience	#14-7325-85	1:500
Biotin- IL-1β	eBioscience	#13-7018-85	1:500
IL-1β	eBioscience	#14-7012-85	1:500
Biotin-IFNγ	eBioscience	#13-7311-85	1:500
IFNγ	eBioscience	#14-7311-85	1:500
IAA	Biorbyt	#orb10922	1:500
**FACS**			
PE-CD4	BD Biosciences	#553653	1:100
FITC-CD25	BD Biosciences	#558689	1:100
APC-Foxp3	eBioscience	#17-5773-80	1:100
FITC-CD8a	BD Biosciences	#553030	1:100
PE-CD8a	Biolegend	#100707	1:100
Alexa Fluor 488-CD11b	BioLegend	# M1/70	1:100
PE-CD11C	BD Biosciences	#557401	1:100
FITC-CD19	BD Biosciences	#557399	1:100
Alexa Fluor® 647- IA/I-E	BD Biosciences	#562367	1:100
APC-IL-6	BD Biosciences	#554400	1:50
APC-IFNγ	BD Biosciences	#554411	1:50
APC-TNFα	BD Biosciences	#11-7349-41	1:100
APC-IL-17	BioLegend	#506915	1:100
Biotin-IL-1β	eBioscience	#13-7018-85	1:100
FITC-CD68 (FA-11)	BioLegend	#137005	1:100
PE-CD3e	BioLegend	#100205	1:100

Alexa 488-CD11b	BioLegend	#101219	1:100
PE-F4/80	BioLegend	#123109	1:100
**ChIP**			
Normal IgG	Santa Cruz	#2027	1:40
NF-κB p65, rabbit	Santa Cruz	#109	1:40
RNA Polymerase II	Millipore	#05-623B	1:500
